# *Mycobacterium tuberculosis *ecology in Venezuela: epidemiologic correlates of common spoligotypes and a large clonal cluster defined by MIRU-VNTR-24

**DOI:** 10.1186/1471-2334-9-122

**Published:** 2009-08-06

**Authors:** Edgar Abadía, Monica Sequera, Dagmarys Ortega, María Victoria Méndez, Arnelly Escalona, Omaira Da Mata, Elix Izarra, Yeimy Rojas, Rossana Jaspe, Alifiya S Motiwala, David Alland, Jacobus de Waard, Howard E Takiff

**Affiliations:** 1 Laboratorio de Genética Molecular, CMBC, Instituto Venezolano de Investigaciones Cientificas (IVIC), 1020A Caracas, Venezuela; 2 Laboratorio de Tuberculosis, Instituto de Biomedicina, Universidad Central de Venezuela, (UCV), Caracas, Venezuela; 3 UCGEI/LaBDEI, Universidad de Carabobo, Valencia, Venezuela; 4 Centro Amazónico de Investigaciones y Control de Enfermedades Tropicales (CAICET), Puerto Ayacucho, Venezuela; 5 Instituto Nacional de Estadistica, (INE), Caracas, Venezuela; 6 Center for Emerging Pathogens, The University of Medicine and Dentistry of New Jersey, Newark, New Jersey, USA

## Abstract

**Background:**

Tuberculosis remains an endemic public health problem, but the ecology of the TB strains prevalent, and their transmission, can vary by country and by region. We sought to investigate the prevalence of *Mycobacterium tuberculosis *strains in different regions of Venezuela. A previous study identified the most prevalent strains in Venezuela but did not show geographical distribution nor identify clonal genotypes. To better understand local strain ecology, we used spoligotyping to analyze 1298 *M. tuberculosis *strains isolated in Venezuela from 1997 to 2006, predominantly from two large urban centers and two geographically distinct indigenous areas, and then studied a subgroup with MIRU-VNTR 24 loci.

**Results:**

The distribution of spoligotype families is similar to that previously reported for Venezuela and other South American countries: LAM 53%, T 10%, Haarlem 5%, S 1.9%, X 1.2%, Beijing 0.4%, and EAI 0.2%. The six most common shared types (SIT's 17, 93, 605, 42, 53, 20) accounted for 49% of the isolates and were the most common in almost all regions, but only a minority were clustered by MIRU-VNTR 24. One exception was the third most frequent overall, SIT 605, which is the most common spoligotype in the state of Carabobo but infrequent in other regions. MIRU-VNTR homogeneity suggests it is a clonal group of strains and was named the "Carabobo" genotype. Epidemiologic comparisons showed that patients with SIT 17 were younger and more likely to have had specimens positive for Acid Fast Bacilli on microscopy, and patients with SIT 53 were older and more commonly smear negative. Female TB patients tended to be younger than male patients. Patients from the high incidence, indigenous population in Delta Amacuro state were younger and had a nearly equal male:female distribution.

**Conclusion:**

Six SIT's cause nearly half of the cases of tuberculosis in Venezuela and dominate in nearly all regions. Strains with SIT 17, the most common pattern overall may be more actively transmitted and SIT 53 strains may be less virulent and associated with reactivation of past infections in older patients. In contrast to other common spoligotypes, strains with SIT 605 form a clonal group centered in the state of Carabobo.

## Background

There has evolved a genre of publications describing aspects of the molecular epidemiology of tuberculosis in varied populations of diverse countries [1-4]. While many of the earlier studies were coupled with epidemiologic investigation to identify routes and risk factors for transmission [5-8], most recent publications describe strain variation and the dynamics in the local population structure of *Mycobacterium tuberculosis*. While this change reflects an increasing interest in the phylogeny of *M. tuberculosis *and its correlation with factors underlying the dynamics in the tuberculosis epidemic, it is also related to the techniques used. Where IS*6110 *RFLP typing distinguishes between strains [9], spoligotyping [10] and sequence polymorphisms [11-13], are suitable for large studies on phylogeny and identification of genotype families. The more recent technique of minisatellite analysis [14], MIRU-VNTR 24 loci, is less technically demanding than the IS*6110 *RFLP typing technique, can discriminate at strain level, and is also promising for use in phylogenetic studies [15]. However, this technique is laborious in its manual form, and fairly expensive when 24 loci are analyzed with an automated sequencer [16]. As spoligotyping is a robust, cheap, and easy-to-perform technique for large scale studies, this is a pragmatic choice, which can be complemented by the selective use of MIRU-VNTR analysis with 24, 15, 12 or fewer loci [17] to discriminate genotypes within clusters of strains showing identical spoligotypes.

In Venezuela, the average national incidence of tuberculosis is moderate, approximately 34 per 100,000 http://www.who.int/globalatlas/predefinedreports/tb/PPDF_Files/ven.pdf. However, the incidence is two to three times higher in some isolated Amerindian populations, likely the result of prolonged deficiencies in control programs, although improvements over the past few years have made strains available for this study. A recent publication described the spoligotype patterns of *M. tuberculosis *strains in Venezuela based on analysis of 670 isolates collected in a nationwide drug resistance survey [18]. While this study identified the predominant spoligotypes in the country, it did not include epidemiologic data, nor information on where in Venezuela each strain was obtained. Also, because it was based on data from spoligotyping alone, it could not discriminate clonal clusters.

**Figure 1:**
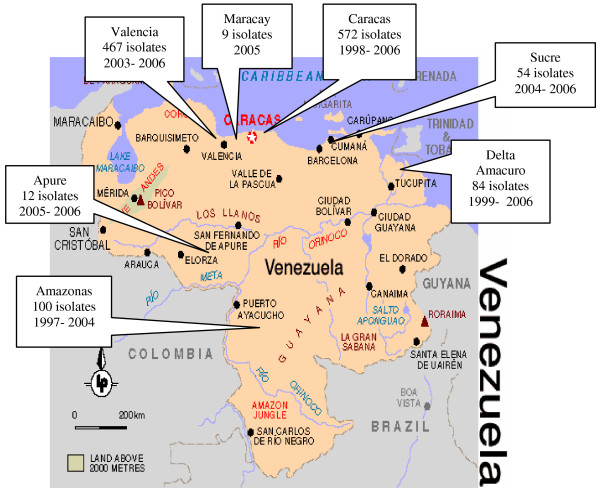
**Map of Venezuela showing the geographic origin of isolates included in the study**.

The Laboratorio de Tuberculosis of the Instituto de Biomedicina and the Laboratorio de Genética Molecular at IVIC, both in Caracas, have been working in collaboration with the National TB Control Program and regional labs in the Amazonas, Carabobo and other states, to implement routine cultures to improve TB diagnosis and control. The *M. tuberculosis *isolates described herein were collected from 1997 through 2006, and while this group of strains could be described as a convenience sample, it is likely to be representative of the country as a whole. It includes large numbers of isolates from the two biggest cities in the center of the country – Caracas and Valencia, as well as from two rural, largely Amerindian populations – Delta Amacuro and Amazonas, located at widely separated geographical poles, and also a smaller number of isolates from other regions (Figure 1 and Additional file 1). Data on strains from the Amazonas [19], Carabobo [20], and Delta Amacuro [21] states that have been described separately but are included here in a broader, national analysis.

The strains were first analyzed by spoligotyping, whereafter a subgroup of strains with the most prevalent spoligotypes were further analyzed with MIRU-VNTR 24 loci typing. We show the geographic distribution of spoligotypes, including a large, geographically centered clonal outbreak, and associate the spoligotype results with epidemiologic parameters to suggest that particular genotypes of *M. tuberculosis *are emerging in Venezuela while others may be disappearing.

## Results

### Clustering Analysis of Spoligotypes

A total of 300 different spoligotype patterns were recognized among the 1298 strains subjected to analysis, of which 201 were unique patterns, and 99 were shared with other isolates, constituting clusters (Additional file 2). Thirty-two clusters comprised only two cases. Of the 300 patterns, 173 were not found in SpolDB4 [10], and of these 32 were shared and 141 were unique types. Of the 127 spoligotypes that have counterparts in SpolDB4, 9 patterns have been found exclusively in Venezuela: ST 1696, belonging to the family LAM5, 21 isolates; ST 1702, LAM5, 10 isolates; ST 375, LAM5, 7 isolates; ST 1692, ×1, 5 isolates; ST 1698, U, 3 isolates; and one isolate each of ST 1104, T5; 1700, T1; ST 1711 LAM2; and ST 1718, X1-LAM9.

**Figure 2:**
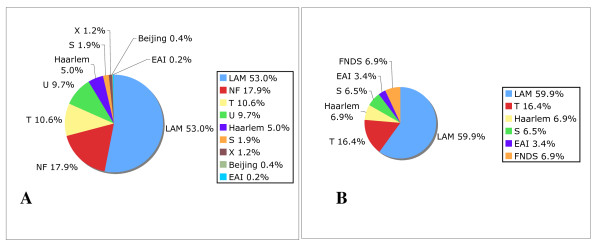
**Distribution of Spoligotype Families**. **A**. Distribution of Venezuelan *M. tuberculosis *biogeographic families defined by SpolDB4. B. Distribution of Venezuelan *M. tuberculosis *spoligotypes absent in SpolDB4 and identified at a family level by SPOTCLUST. NF= Not found in SpolDB4. FNDS= Family not described in SpolDB4.

The most common spoligotype families were LAM (53%), T (10.6%), and Haarlem (5%), but 17.9% could not be classified (Figure 2). Using the program SPOTCLUST to predict the most likely family, a similar distribution was obtained for the 32 cluster spoligotypes (91 isolates) not found in SpolDB4 (Additional file 2).

**Figure 3:**
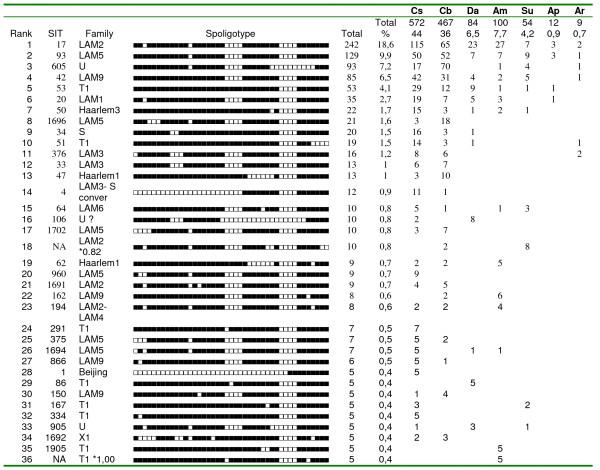
**Geographic Distribution of the Most Common Spoligotypes in Venezuela**. Spoligotypes found in at least 5 isolates, ranked by number of isolates, with SIT, Spoligotype family (SpoDB4), geographic distribution, and percentage of total strains (1298). Cs = Caracas, Cb= Carabobo State, Da = Delta Amacuro State, Am = Amazonas State; Suc = Sucre State, Ap = Apure State, and Ar = Aragua State. For spoligotypes not represented in SpolDB4, the "*" next to the family designation indicates the probability of belonging to that family as determined by the program SPOTCLUST.

The six most common spoligotypes (Figure 3) were Shared Types (SIT's) 17, 93, 605, 42, 53 and 20, which together accounted for 49% of the total isolates. The single most common pattern, SIT 17, was found in 242 isolates (18.6%). The most common spoligotypes overall were also the most common in each region, with SIT 17 being the first or second most frequently isolated in all regions studied.

Although SIT 605 is relatively uncommon in most regions, it was the third most common spoligotype overall because the collection includes 467 strains (36% of the total) from the state of Carabobo, where it is the most common pattern. It is present in SpolDB4, but the only two strains with SIT605 described outside of Venezuela were isolated in New York in patients from Colombia (Jeffrey Driscoll, Natalia Kurepina and Barry Kreiswirth, personal communication), which shares a long border with Venezuela.

In SpolDB4, SIT 605 is not clearly assigned to a major genotype family, but the absence of spacers 21 to 24 and 33 to 36 would place it in the LAM family, even though it also lacks spacers 31 – 32 and 37 – 40. To confirm its designation as LAM, 45 chromosomal SNPs [13] were analyzed for 23 strains, including 4 SIT 605, 4 SIT 17 and 4 SIT 93. SIT 605 was found to belong to SCG v-ST7, as were the SIT 17, SIT 93 strains, as well as the other 11 strains with different LAM spoligotypes (data not shown). If SIT 605 is classified as a LAM strain, this family would account for a total of 60.2% of the isolates in this study.

### MIRU-VNTR 24 Loci Analysis

**Figure 4:**
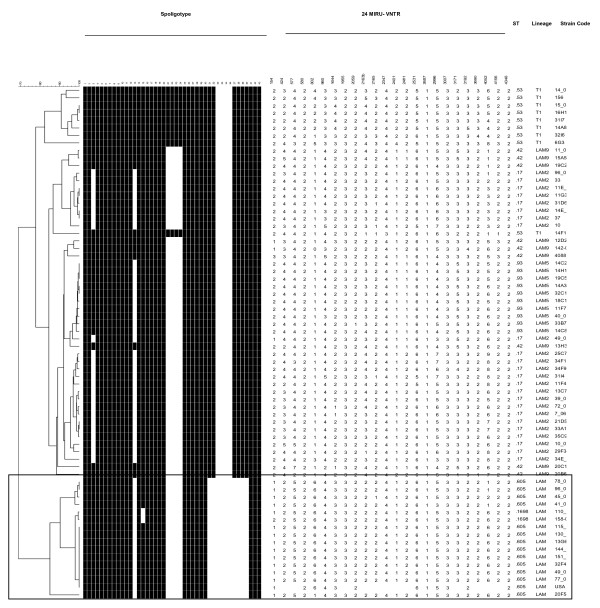
**Dendrogram of Venezuelan *M. tuberculosis *strains**. Dendrogram combining MIRU-VNTR and Spoligotype results from representative strains generated by Bionumerics software (Applied Maths) using the unweighted pair group method of averages (UPGMA). MIRU patterns with 3 or fewer changes are considered clonal. Inside the box are the SIT 605 strains defined as clonal by spoligo and by 24 MIRU- VNTR loci. Only MIRU 12 results were available for the strains of SIT 605 isolated in New York.

To determine the genotype similarity of strains sharing identical spoligotype patterns, we performed MIRU-VNTR 24 loci analysis on a subgroup of strains representing the major spoligotype clusters (Figure 4). MIRU-VNTR confirmed the spoligotyping designation in all strains. Surprisingly, while only occasional strains with SIT 17 or SIT 93 shared MIRU-VNTR 24 patterns, all SIT 605 strains were identical for at least 21 loci, even if the strains had been isolated at distinct geographic regions, suggesting that SIT 605 comprises a clonal group of strains. Even the two SIT 605 strains isolated in New York also appeared to be part of this clone, based on previous MIRU 12 results (Jeffrey Driscoll personal communication). We propose naming this strain the "Carabobo" genotype. Furthermore, ST 1698, whose spoligotype pattern differs from SIT 605 by the absence of one additional spacer, was found by MIRU-VNTR 24 to belong to the SIT 605 "Carabobo" genotype.

### Correlation with Epidemiologic Parameters

Epidemiologic data was not available for all of the patients from whom the 1298 strains had been isolated, but for several parameters the information was recorded for at least 25% of the 1298 patients. Overall, of those patients with available data, the mean age was 38 years, 68% were between 15 and 45 years of age, while 29% were over 45, and just 3% were less than 15 years old. Sixty nine percent were men, 74% had a positive clinical specimens, 88% had pulmonary disease, and 75% had cavitations according to the chest X-rays (data not shown). Venezuelans made up 93% of patients and foreign-borns just 7%. The distribution of spoligotype patterns in isolates from the foreign born population did not appear different from that of native Venezuelan patients.

Comparisons of patients in different regions revealed that the average patient age was similar for the two urban areas – 39 years for Carabobo and 37.8 years for Caracas – and 36.8 years for the Amazon region (Table 1). However it was much younger, 32.6 years, for patients from the indigenous Delta Amacuro region. Also, while males made up about 71% of patients from Caracas, 70% from Carabobo (69%) [20], and 64% from Amazonas [19], males comprised only 54% of the patients from Delta Amacuro [21]. Surprisingly, female patients had significantly lower mean ages overall, and also in Caracas and in Delta Amacuro (Table 1).

**Table 1:** Comparison of mean ages of TB patients whose isolates were included in this study

	Overall	Amazonas	Caracas	Carabobo	Delta Amacuro	Sucre	Apure
N° of individuals	1298	100	572	467	84	54	12
Individuals with age data	766	100	410	212	22	15	7
Mean age (years)	38.0 (37–39)	36.8 (33–41)	37.8 (36–39)	40.0 (37–41)	31.2 (23–39)	44.7 (34–55)	42.6 (27–58)
N° of females	304	36	151	66	33	11	4
% Females	31	36	29	30	46	24	33
Female with age data	241	36	118	66	13	6	2
Mean Female age (years)	36.2 (34–38)	38.5 (32–45)	35.1 (32–38)	37.6 (33–42)	24.5 (15–34)	48.8 (30–67)	
N° of males	670	64	373	157	38	34	8
% Males							
Males with age data	525	64	292	156	9	9	5
Mean Male Age	38.8 (37–40)	35.8 (31–41)	38.8 (37–41)	39.6 (37–42)	40.9 (28–54)	42.0 (30–54)	
Difference M/F Mean age p value	0.05	0.52	0.03	0.39	0.05	0.54	

The mean ages are shown for the entire group and also separated by region and gender. In each category, the age was available only for the number of patients indicated with age data. Confidence intervals are shown in parentheses. Determination of mean ages, confidence intervals and statistical significance using the Student's two -tailed T test assuming equal variance were performed within Excel. Statistically significant *p *values are shown in bold.

There were no significant differences when all patients in clusters were compared with all patients with non-clustered isolates. However, Table 2 shows that patients whose isolates had the most common spoligotype, SIT 17, were significantly younger compared to patients with other spoligotype strains (mean ages 34 vs 39 years, p = 0.0008). The SIT 17 patients also had lower mean ages when the comparisons were stratified for each of the three regions with the most isolates, but the differences did not quite reach statistical significance (Caracas 35 vs 38.4, p = 0.077; Carabobo 34.5 vs 39.8 p = 0.076; and Amazonas 30.6 vs 39.1, p = 0.056). In contrast, patients with SIT 53 had a significantly higher mean age compared to all other patients (48.2 vs 37.6, p = 0.001), or compared within the Caracas cohort (50.25 vs 37.1, p = 0.0002), the source of most SIT 53 patients with epidemiologic data. The mean ages for patients in the other large clusters weren't significantly different from the overall group (data not shown).

**Table 2:** Comparison of mean ages of TB patients with SIT 17 or SIT 53 spoligotype patterns

	Overall	Caracas	Carabobo	Amazonas	Delta Amacuro	Sucre	Apure
N° of isolates	1298	572	467	100	84	54	12
% with age	59	72	45	100	25	28	58
Mean age	37.6	37.8	39.0	36.8	32.6	44.7	42.6
	(36–39)	(36–39)	(37–41)	(33–41)	(24–41)	(34–55)	(27–58)

SIT 17	242	115	65	27			
% with age	64	66	52	100			
Mean age	34	35	34	31			
	(32–36)	(32–38)	(30–39)	(24–37)			

Non SIT 17	1056	457	402	73			
% with age	58	73	44	100			
Mean age	39	38	40	39			
	(38–40)	(37–40)	(37–42)	(34–44)			
17 vs non 17	P = 0.0008	P = 0.077	P = 0.076	P = 0.056			

SIT 53	53	29					
% with age	42	69					
Mean age	48	50					
	(42–57)	(42–58)					

Non SIT 53	1245	544					
% with age	60	72					
Mean age	38	37					
	(36–39)	(36–39)					
53 vs non 53	P = 0.001	P = 0.0002					
Non 53 non 17	1004	413					
% with age	59	76					
Mean age	38.63	37.7					
	(37–40)	(36–39)					
53 vs non 53 non 17	P = 0.003	P = 0.0005					

The table shows the mean age of all patients with age data in the study as a single cohort and also divided by region. Statistical comparisons are shown between patients with SIT 17 and patients with other spoligotypes and, separately, between SIT 53 and other spoligotypes. Confidence intervals are shown in parentheses. Mean values, confidence intervals, and P values with the Student's two-tailed T test assuming equal variance were performed within Excel. Statistically significant *p *values are shown in bold.

As seen in Table 3, patients with SIT 17 isolates were also more likely to have had clinical specimens positive for Acid Fast Bacilli (AFB) by microscopy when compared overall, or just within the Caracas cohort, but the differences were only significant compared to other Caracas patients (SIT 17 vs non-SIT 17, all patients: OR = 1.47, p = 0.14; Caracas: OR = 1.96, p = 0.04). Within the Caracas group, specimens from patients with the SIT 53 spoligotype were less likely be AFB positive, but the difference was not statistically significant (OR = 0.68, p = 0.55). However, there were only 20 Caracas SIT 53 patients with AFB microscopy results. The difference was more marked when AFB positivity was compared between Caracas patients with SIT 17 and SIT 53, but still failed to reach statistical significance (OR = 2.44, p = 0.14).

**Table 3:** Comparison of specimens whose cultures grew isolates with SIT 17 or SIT 53 for percentage that were positive for Acid Fast Bacilli by microscopy

	All	All w/o SIT 17	All SIT 17	CCS all	CCS w/o SIT 17	CCS SIT 17	CCS w/o SIT 53	CCS SIT 53
Total	1298	1054	244	572	457	115	543	29
% with data	48	48	50	53	53	51	52	69
Positive	463	366	97	178	136	42	168	10
Negative	164	139	25	125	108	17	115	10
% positive	74	72	80	59	56	71	59	50
		All:		CCS:		CCS:		CCS:
Comparison		17 vs non 17		17 vs non 17		53 vs non 53		17 vs 53
Odds ratio		OR= 1.47		OR= 1.96		OR= 1.46		OR= 2.44
		(0.91–2.43)		(1.06–3.7)		(0.57–3.7)		(0.84–7.11)
		P = 0.14		P = 0.04		P = 0.55		P = 0.14

Comparisons were made based on the patients with microscopy data. The percentage of specimens that were AFB positive was compared between those that grew strains with either spoligotype SIT 17 or SIT 53, and those from which strains with all other patterns were isolated. Comparisons were made for all specimens in the study, and separately, for those obtained in Caracas (CCS). Two by two table comparisons with odds ratios and Yates corrected two-tail chi square values were calculated using OpenEpi http://www.openepi.com. Statistically significant *p *values are shown in bold

## Discussion

We have used Spoligotyping to analyze *M. tuberculosis *strains isolated in different parts of Venezuela, and while the collection could be described as a convenience sample, without proportionate geographic or temporal distribution, it likely reflects the prevalence of strains in the entire country, as the spoligotypes are generally the same as those found in a previous study based on a proportional nationwide sampling to determine prevalence of drug resistance [18]. There are a total of approximately 6500 cases of tuberculosis reported per year in Venezuela, so for the ten years during which strains were collected, the sample represents only ~2% of registered cases, but 5% for Caracas and a larger percentage for Valencia and the Carabobo state of which it is the capital.

As in other studies in South America [18,22,23] the LAM family predominates, accounting for 53% of all strains, and 60% if, as suggested by the SNP results, SIT 605 is also considered LAM. Although 300 different spoligotype patterns were found, only 6 accounted for almost 50% of the isolates, and these patterns were the most common in nearly all the regions sampled. In contrast, 201 patterns were found in only one isolate, and 173 (57.7%) of these were not present in SpolDB4. Nine of the 127 patterns that are represented in SpolDB4 have been found exclusively in Venezuela, and a tenth, SIT 605, has been isolated outside of Venezuela only in New York, in two immigrants from the neighboring country of Colombia. This last spoligotype, SIT 605, is perhaps the most surprising finding in this study, because unlike the other common spoligotypes such as SIT 17 and 93, which contain a number of distinct genotypes when analyzed by MIRU-VNTR 24 loci, SIT 605 appears to represent a large clonal cluster that is geographically centered around the city of Valencia and the state of Carabobo [20], but encompasses almost all the SIT 605 strains examined, including the two isolated in New York. The previous study [18] of the molecular epidemiology of *M. tuberculosis *strains in Venezuela found several isolates with SIT 605, but as that study was based on spoligotyping without geographic data, the focal and clonal nature of this genotype could not have been detected. We propose that the SIT 605 strains be termed the "Carabobo" genotype.

Phylogeographic analysis of spoligotypes have identified a number of regionally originating patterns or clades whose isolates have similar patterns with RFLP IS*6110 *and appear to represent strains with a common lineage. The most notorious example is SIT 1, or Beijing, which presumably originated in Asia and has spread widely [24]. Other examples are the SIT 33/F11/LAM clade [25] first described in South Africa, the SIT 60/F15/LAM4 KZN clade associated with XDR-TB in Kwazulu Natal, South Africa [26], the SIT 61/LAM10 Cameroon clade, SIT 59/LAM11/ZWE/MERU, SIT 21/CAS1-Kili, SIT 26/CAS1-Dehli, SIT 41/LAM7-Tur, and SIT 19/Manila/EAI2 [10,27]. These clades are not homogeneous, but include several related MIRU-VNTR and RFLP IS*6110 *patterns, some of which may comprise more localized clonal clusters. However, the fact that these spoligotype families have a wide distribution, and some are associated with MDR outbreaks, suggests that they have selective advantages over other strains in their ability to cause disease and be transmitted. It appears that SIT 17, and perhaps SIT 93, might similarly be considered successful clades, as they are responsible for 18.6% and 9.9%, respectively, of tuberculosis cases in Venezuela, and have been found in several other countries.

There are also reports of spoligotype patterns that are geographically limited and appear to be clonal by MIRU/RFLP analyses, such as the SIT 2643 Haarlem3 strain in Paraguay [22], but the number of isolates of these clonal genotypes is generally small. An examination of SpolDB4 [10] shows several SIT's with more than 10 isolates limited to one country, or with a single additional isolate in a separate country, perhaps from an emigrant (for example, SIT's 210, 339, 1258, 1329, 1457, 1518, and 1898). Similarly, in Additional file 2 there are, besides SIT 605, other spoligo patterns that could represent clonal strains because they are found only in particular regions of Venezuela, for example the putative LAM2 strain in position 18 (Figure 3). Several of these are currently being analyzed by MIRU-24 loci. However, it is striking that the SIT 605/SIT 1698 Carabobo genotype is the most common spoligotype in a state with a population greater than 2 million, accounting for 15% of all isolates, while it is relatively uncommon in the other regions we sampled. However, of only nine isolates obtained from the state of Aragua, which borders Carabobo, one was SIT 605, so it is possible that this strain is also common in adjacent areas. As the spoligotype for the Carabobo strain evolved with the loss of spacers 31–32 and 37 – 40, other elements of the genome could have, independently, evolved to gain an unknown selective advantage over other local *M. tuberculosis *genotypes, but unlike SIT 17, there were no epidemiologic correlations suggesting higher virulence. At least six SIT 605 patients were present or former inmates in a particular prison in Carabobo state, and it appears that other SIT 605 patients had contact with people that were interned in that prison, so this institution could play a role in its local dissemination, as described for other prison-associated clonal outbreaks [28,22,29].

Although at least one SIT 605 isolate was found to be Multi-Drug Resistant [18], the great majority of SIT 605 isolates were pan-sensitive. Our study found only a very few Beijing isolates, some of them lethal MDR strains (National Tuberculosis Program, unpublished data), but fortunately they do not appear to have been transmitted extensively within the Venezuelan population [30]. At least one of the Beijing strains was isolated from a Peruvian national [31].

The comparisons of epidemiologic and clinical parameters revealed that in the indigenous Warao population in Delta Amacuro [21] tuberculosis occurs at younger ages (mean of 32.6 years) than in the rest of the population (mean of 38–39 years), and unlike the 71% male predominance in the cities, there is a nearly equal male:female distribution (54%). This pattern is consistent with very active spread within this isolated Amerindian community [21] that has the highest TB incidence in Venezuela and amongst the lowest life expectancies. The data from the largely Amerindian tuberculosis population in the Amazonas states [19] suggests a similar, but less marked trend, with an average age of 36.8 that is 64% male. More polemical are the findings suggesting that SIT 17 may be more actively transmitted because patients with isolates belonging to the SIT 17 cluster tended to be younger and were more likely to have AFB positive specimens. Low patient age is considered characteristic of strains being actively transmitted [32]. In contrast, SIT 53 may be less virulent because patients with this spoligotype tended to be older and may have more AFB negative sputa, but the AFB trend did not reach statistical significance, and the older age, while reaching statistical significance, was based on only 21 SIT 53 patients and needs to be confirmed in subsequent studies.

These associations provoke a number of questions. If SIT 53 were really less virulent, why is it still the sixth most common spoligotype, causing 4% (53) of cases? Could it have been a very common strain in the past, that is now more apt at latency and reactivation than person-to-person transmission, and will its prevalence decrease over time? SIT 93 is the second most common spoligotype, found in 10% of all isolates, but why isn't it also associated with clinical parameters suggesting virulence and transmission? Finally, preliminary MIRU-VNTR analysis suggests that all of the most common spoligotype clusters, except for SIT 605, contain several different strain genotypes. Therefore, for the epidemiologic associations with SIT 17 or SIT 53 to make sense, it must be assumed that the strains with these spoligotypes derived from a common ancestor with genetic characteristics that were maintained even as the MIRU-VNTR patterns evolved, as seems true for globally dispersed lineages such as Beijing. Finally, it must be recalled that clinical data was not available for many of the patients whose isolates comprised this study, so although all data were collected before spoligotyping results were known, the epidemiologic associations with SIT 17 could be subject to biases due to regional differences in patient admission or data recording. We attempted to reduce this possibility through regional stratification, and found that associations for SIT 17 persisted, although not always reaching statistical significance. If subsequent studies with more complete data confirm that the SIT 17 or SIT 53 spoligotypes, or the SIT 605 Carabobo genotype, are associated with particular clinical disease characteristics, the challenge will be to identify the molecular basis for these apparent differences.

## Conclusion

Six spoligotypes accounted for 49% of the 1298 *M. tuberculosis *Venezuelan isolates analyzed, and the most common overall were the most common in all regions examined. The patients with isolates showing spoligotype SIT 17 tended to be younger and more likely to have had AFB positive specimens, suggesting that these strains are being actively transmitted. In contrast, SIT 53 may be more common in cases of reactivation, as the patients with these strains tended to be older and may be less likely to be AFB positive. MIRU-VNTR 24 showed that only a minority of strains in the most common spoligotypes were clustered, with the exception of SIT 605 strains, which appear to be clonal. SIT 605 was named the "Carabobo" genotype because it is the most common spoligotype in this state, but infrequently seen in other regions of Venezuela. The only two strains with SIT 605 described outside of Venezuela were isolated in New York in patients from Colombia, and also belong to the SIT 605 cluster. Overall, female patients had lower mean ages than male patients. Tuberculosis patients in the Amerindian Warao population in the state of Delta Amacuro had a lower mean age and a more equal male:female distribution, possibly reflecting the active transmission that gives this population the highest incidence of tuberculosis in the country.

## Methods

### *M. tuberculosis *strains

The 1298 clinical isolates in this report were obtained from an equal number of different patients between 1997 and 2006 (Additional file 1) as part of an ongoing study of *M. tuberculosis *strains from different geographic regions of Venezuela (Figure 1). They are largely from two large urban regions, Caracas (pop. > 5 million), the national capital (*n *= 572), and Valencia (pop. >1.5 million), capital of the state of Carabobo (Pop. > 2.2 million) (*n *= 467), and two relatively isolated states with large and distinct Amerindian populations, Delta Amacuro – where the Warao inhabit the wide delta of the Orinoco River (*n *= 84) and Amazonas (*n *= 100), home to many Amerindian ethnicities. Smaller numbers of strains were also obtained from the states of Apure (*n *=12), Aragua (*n *= 9) and Sucre (*n *= 54). The isolates were obtained by culturing sputa or non-pulmonary clinical specimens using either the Petroff method with Löwenstein – Jensen slants or the Kudoh method with Ogawa slants [33] The criteria for culturing clinical specimens varied by region. All that were positive for Acid Fast Bacilli by microscopy were cultured, but some regions, such as Carabobo, also cultured specimens from patients that were symptomatic and had abnormal chest X-rays or were otherwise highly suspicious for having TB. In Caracas all specimens from patients with respiratory symptoms for more than two weeks were cultured.

### Spoligotyping

Spoligotyping was performed as previously described by Kamerbeek [34]. The spacer sequences contained in the direct repeat locus were detected by hybridization onto either homemade or commercially prepared spoligotyping membranes. DNAs from *M. tuberculosis *H37Rv and *M. bovis *BCG were used as positive controls, and spoligotyping was repeated until results were unambiguous. Each spoligotype was converted to octal format [35] and compared with the International Spoligo Database, SpolDB4 [10]. SPOTCLUST, an on-line program developed by Vitol *et al*. [36], was used to tentatively assign phylogeographic families to spoligotype patterns not present in SpolDB4.

Patient data, including age, sex, nationality, HIV status, AFB positivity of the original clinical specimen, treatment history, and chest X-ray information were included, when available, in the spreadsheets that contained the spoligotyping results.

### MIRU-VNTR Genotyping

Twenty four loci were amplified using multiplex PCR and run with Rox labeled Map Marker 1000 size standards (Bioventures) on ABI 3130 xl sequencers as described [14,16,37,38]. Sizing of the PCR fragments and assignments of MIRU-VNTR alleles were done with the Gene Mapper software package (PE Applied Biosystems).

### Cluster analysis

Spoligotyping and MIRU-VNTR profiles were also recorded as character data and analyzed using Bionumerics software (Applied Maths) or MIRU-VNTR *plus*, an on line program developed by Allix-Béguec et al [39]. Dendrograms were generated by using the categorical character option and the unweighted pair group method of averages (UPGMA) clustering method.

### SNP Analysis

A subset of strains was analyzed for a set of 45 informative SNPs as described [13].

### Statistical analysis of epidemiologic data

The clinical and epidemiologic characteristics of patients belonging to each of the six most common spoligotype clusters were compared between these clusters and also against all patients not in the analyzed cluster. Comparisons of sputum positivity were done as two by two tables using the Yates corrected two-tail chi-square test on the OpenEpi website http://www.openepi.com. Mean age calculations, confidence intervals and age comparisons with the two-tailed T Test, assuming equal variance, were performed within Excel. A *p *value of < 0.05 was considered statistically significant.

## Competing interests

The authors declare that they have no competing interests.

## Authors' contributions

EA performed and analyzed spoligotyping, analyzed epidemiologic data and helped draft the manuscript; MS isolated strains, collected epidemiologic data, and performed spoligotyping; DO isolated strains and collected epidemiologic data; MVM performed and analyzed MIRU-VNTR-24; AE and ODM isolated strains and performed spoligotyping; YR and RJ isolated strains, performed and analyzed spoligotyping; ASM and DA performed and analyzed SNP studies; EI helped with the statistical analysis of the epidemiologic data; JdW directed strain isolation and spoligotyping and helped to conceive the study; HET conceived and directed the study, analyzed the data and wrote the manuscript.

## Pre-publication history

The pre-publication history for this paper can be accessed here:

http://www.biomedcentral.com/1471-2334/9/122/prepub

## Supplementary Material

Additional file 1 **Distribution of strains included in the study by year and region of isolation**. Cs = Caracas, Cb = Carabobo State, Da = Delta Amacuro State, Am = Amazonas State; Suc = Sucre State, Ap = Apure State, and Ar = Aragua State. Click here for file

Additional file 2 **All Spoligotypes found in the study**. Spoligotypes found in the study, ranked by number of isolates, with SIT, Spoligotype family (SpoDB4), geographic distribution, and percentage of total (1298). Cs = Caracas, Cb = Carabobo State, Da = Delta Amacuro, Am = Amazonas State; Suc = Sucre, Ap = Apure, and Ar = Aragua state. The "*" next to a family designation indicates the probability of belonging to that family as determined by the program SPOTCLUST. Click here for file
