# Supplementary material for: Mycobacterium tuberculosis ecology in Venezuela: epidemiologic correlates of common spoligotypes and a large clonal cluster defined by MIRU-VNTR-24
Source: BMC Infect Dis. 2009 Aug 6;9:122. doi: 10.1186/1471-2334-9-122 (PMC2739208; doi:10.1186/1471-2334-9-122)
Supplement: Additional file 1 — Distribution of strains included in the study by year and region of isolation. Cs = Caracas, Cb = Carabobo State, Da = Delta Amacuro State, Am = Amazonas State; Suc = Sucre State, Ap = Apure State, and Ar = Aragua State. [file 1471-2334-9-122-S1.doc]

Supplementary Table 1.

| **Region** | **1997** | **1998** | **1999** | **2000** | **2001** | **2002** | **2003** | **2004** | **2005** | **2006** | **Total** | **%** |
| --- | --- | --- | --- | --- | --- | --- | --- | --- | --- | --- | --- | --- |
| **Cs** |  | 1 | 64 | 26 | 70 | 88 | 138 | 47 | 55 | 78 | 567 | 44 |
| **Cb** |  |  |  |  |  |  | 62 | 157 | 144 | 104 | 467 | 36 |
| **Da** |  |  | 70 |  |  | 1 | 5 | 3 |  | 4 | 83 | 6.5 |
| **Pa** | 2 | 5 | 15 | 7 | 1 | 35 | 18 | 17 |  |  | 100 | 7.7 |
| **Suc** |  |  |  |  |  |  |  | 10 | 38 | 6 | 54 | 4.2 |
| **Ap** |  |  |  |  |  |  |  |  | 1 | 11 | 12 | 0.9 |
| **Ara** |  |  |  |  |  |  |  |  | 9 |  | 9 | 0.7 |
| **Total** | 2 | 6 | 149 | 33 | 71 | 124 | 223 | 234 | 247 | 203 | **1292** |  |
| % | 0.2 | 0.5 | 11.5 | 2.6 | 5.5 | 9.6 | 17.3 | 18.1 | 19.1 | 15.7 |  |  |
