# Supplementary material for: Mycobacterium tuberculosis ecology in Venezuela: epidemiologic correlates of common spoligotypes and a large clonal cluster defined by MIRU-VNTR-24
Source: BMC Infect Dis. 2009 Aug 6;9:122. doi: 10.1186/1471-2334-9-122 (PMC2739208; doi:10.1186/1471-2334-9-122)
Supplement: Additional file 2 — All Spoligotypes found in the study. Spoligotypes found in the study, ranked by number of isolates, with SIT, Spoligotype family (SpoDB4), geographic distribution, and percentage of total (1298). Cs = Caracas, Cb = Carabobo State, Da = Delta Amacuro, Am = Amazonas State; Suc = Sucre, Ap = Apure, and Ar = Aragua state. The "*" next to a family designation indicates the probability of belonging to that family as determined by the program SPOTCLUST. [file 1471-2334-9-122-S2.doc]

Supplementary Table 2.

|  |  |  |  |  |  |  | **Cs** | **Cb** | **Da** | **Am** | **Su** | **Ap** | **Ar** |
| --- | --- | --- | --- | --- | --- | --- | --- | --- | --- | --- | --- | --- | --- |
|  |  |  |  |  |  | # | 572 | 467 | 84 | 100 | 54 | 12 | 9 |
| **Rank** | **SIT** | **Family** | **Spoligotype** | **Octal Code** | **Total** | **%** | 44 | 36 | 6,5 | 7,7 | 4,2 | 0,9 | 0,7 |
| 1 | 17 | LAM2 | ■■□■■■■■■■■■□■■■■■■■□□□□■■■■■■■■□□□□■■■■■■■ | 677737607760771 | 242 | 18,6 | 115 | 65 | 23 | 27 | 7 | 3 | 2 |
| 2 | 93 | LAM5 | ■■■■■■■■■■■■□■■■■■■■□□□□■■■■■■■■□□□□■■■■■■■ | 777737607760771 | 129 | 9,9 | 50 | 52 | 7 | 7 | 9 | 3 | 1 |
| 3 | 605 | U | ■■■■■■■■■■■■□■■■■■■■□□□□■■■■■■□□□□□□□□□□■■■ | 777737607700031 | 93 | 7,2 | 17 | 70 |  | 1 | 4 |  | 1 |
| 4 | 42 | LAM9 | ■■■■■■■■■■■■■■■■■■■■□□□□■■■■■■■■□□□□■■■■■■■ | 777777607760771 | 85 | 6,5 | 42 | 31 | 4 | 2 | 5 |  | 1 |
| 5 | 53 | T1 | ■■■■■■■■■■■■■■■■■■■■■■■■■■■■■■■■□□□□■■■■■■■ | 777777777760771 | 53 | 4,1 | 29 | 12 | 9 | 1 | 1 | 1 |  |
| 6 | 20 | LAM1 | ■■□■■■■■■■■■■■■■■■■■□□□□■■■■■■■■□□□□■■■■■■■ | 677777607760771 | 35 | 2,7 | 19 | 7 | 5 | 3 |  | 1 |  |
| 7 | 50 | Haarlem3 | ■■■■■■■■■■■■■■■■■■■■■■■■■■■■■■□■□□□□■■■■■■■ | 777777777720771 | 22 | 1,7 | 15 | 3 | 1 | 2 | 1 |  |  |
| 8 | 1696 | LAM5 | ■■■□□□■■■■■■□■■■■■■■□□□□■■■■■■■■□□□□■■■■■■■ | 707737607760771 | 21 | 1,6 | 3 | 18 |  |  |  |  |  |
| 9 | 34 | S | ■■■■■■■■□□■■■■■■■■■■■■■■■■■■■■■■□□□□■■■■■■■ | 776377777760771 | 20 | 1,5 | 16 | 3 | 1 |  |  |  |  |
| 10 | 51 | T1 | ■■■■■■■■■■■■■■■■■■■■■■■■■■■■■■■■□□□□■■■□□□□ | 777777777760700 | 19 | 1,5 | 14 | 3 | 1 |  |  |  | 1 |
| 11 | 376 | LAM3 | □■■■■■■■□□□■■■■■■■■■□□□□■■■■■■■■□□□□■■■■■■■ | 376177607760771 | 16 | 1,2 | 8 | 6 |  |  |  |  | 2 |
| 12 | 33 | LAM3 | ■■■■■■■■□□□■■■■■■■■■□□□□■■■■■■■■□□□□■■■■■■■ | 776177607760771 | 13 | 1 | 6 | 7 |  |  |  |  |  |
| 13 | 47 | Haarlem1 | ■■■■■■■■■■■■■■■■■■■■■■■■■□□□□□□■□□□□■■■■■■■ | 777777774020771 | 13 | 1 | 3 | 10 |  |  |  |  |  |
| 14 | 4 | LAM3- S conver | □□□□□□□□□□□□□□□□□□□□□□□□■■■■■■■■□□□□■■■■■■■ | 000000007760771 | 12 | 0,9 | 11 | 1 |  |  |  |  |  |
| 15 | 64 | LAM6 | ■■■■■■■■■■■■■■■■■■■■□□□□■■■■□■■■□□□□■■■■■■■ | 777777607560771 | 10 | 0,8 | 5 | 1 |  | 1 | 3 |  |  |
| 16 | 106 | U (U/LAM3)? | ■■■■■■■■□□□■■■■■■■■□□□□□□□□□□□□□□□□□□□■■■■■ | 776177400000171 | 10 | 0,8 | 2 |  | 8 |  |  |  |  |
| 17 | 1702 | LAM5 | □□□□■■■■■■■■□■■■■■■■□□□□■■■■■■■■□□□□■■■■■■■ | 037737607760771 | 10 | 0,8 | 3 | 7 |  |  |  |  |  |
| 18 | NA | LAM2 *^0.82^ | ■■□■■■■■■■■■□■■■■■■■□□□□■■■■■□□■□□□□■■■■■□□ | 677737607620760 | 10 | 0,8 |  | 2 |  |  | 8 |  |  |
| 19 | 62 | Haarlem1 | ■■■■■■■■■■■■■■■■■■■■■■■■■□□□□□□■□□□□■■■□■■■ | 777777774020731 | 9 | 0,7 | 2 | 2 |  | 5 |  |  |  |
| 20 | 960 | LAM5 | ■□□■■■■■■■■■□■■■■■■■□□□□■■■■■■■■□□□□■■■■■■■ | 477737607760771 | 9 | 0,7 | 9 |  |  |  |  |  |  |
| 21 | 1691 | LAM2 | ■■□■■■■■■■■■□■□■■■■■□□□□■■■■■■■■□□□□■■■■■■■ | 677727607760771 | 9 | 0,7 | 4 | 5 |  |  |  |  |  |
| 22 | 162 | LAM9 | ■■■■■■■■■■■■■■■■■■■■□□□□■■■■■■■■□□□□■■□■■■■ | 777777607760671 | 8 | 0,6 |  | 2 |  | 6 |  |  |  |
| 23 | 194 | LAM2-LAM4 | ■■□■■■■■■■■■□■■■■■■■□□□□■■■■■■■■□□□□■■■□■■■ | 677737607760731 | 8 | 0,6 | 2 | 2 |  | 4 |  |  |  |
| 24 | 291 | T1 | ■■■■■■■■■■■■■■■■■■■■□■■■■■■■■■■■□□□□■■■■■■■ | 777777677760771 | 7 | 0,5 | 7 |  |  |  |  |  |  |
| 25 | 375 | LAM5 | □□□■■■■■■■■■□■■■■■■■□□□□■■■■■■■■□□□□■■■■■■■ | 77737607760771 | 7 | 0,5 | 5 | 2 |  |  |  |  |  |
| 26 | 1694 | LAM5 | □■□■■■■■■■■■□■■■■■■■□□□□■■■■■■■■□□□□■■■■■■■ | 277737607760771 | 7 | 0,5 | 5 |  | 1 | 1 |  |  |  |
| 27 | 866 | LAM9 | ■□■■■■■■■■■■■■■■■■■■□□□□■■■■■■■■□□□□■■■■■■■ | 577777607760771 | 6 | 0,5 | 5 | 1 |  |  |  |  |  |
| 28 | 1 | Beijing | □□□□□□□□□□□□□□□□□□□□□□□□□□□□□□□□□□■■■■■■■■■ | 000000000003771 | 5 | 0,4 | 5 |  |  |  |  |  |  |
| 29 | 86 | T1 | ■■■■■■■■■■■■■■■■■■■■■□■■■■■■■■■■□□□□■■■■■■■ | 777777737760771 | 5 | 0,4 |  |  | 5 |  |  |  |  |
| 30 | 150 | LAM9 | ■■■■■■■■■■■■■■□■■■■■□□□□■■■■■■■■□□□□■■■■■■■ | 777767607760771 | 5 | 0,4 | 1 | 4 |  |  |  |  |  |
| 31 | 167 | T1 | ■■■■■■■■■■■■■■■■■■■■■■■■■■■■■□■■□□□□■■■■■■■ | 777777777660771 | 5 | 0,4 | 3 |  |  |  | 2 |  |  |
| 32 | 334 | T1 | ■□■■■■■■■■■■■■■■■■■■■■■■■■■■■■■■□□□□■■■■■■■ | 577777777760771 | 5 | 0,4 | 5 |  |  |  |  |  |  |
| 33 | 905 | U | ■■□■■■■■■■■■□■■■■■■■□□□□■■■■■■□□□□□□■■■■■■■ | 677737607700771 | 5 | 0,4 | 1 |  | 3 |  | 1 |  |  |
| 34 | 1692 | X1 | ■□□□□■■■■□□■□■■■■□■■■■■■■■■■■■■■□□□□■■■□■■■ | 417136777760731 | 5 | 0,4 | 2 | 3 |  |  |  |  |  |
| 35 | 1905 | T1 | ■■■■■■■■■■■■■■■■■■■■■■■■■■■■□□■■□□□□■■■■■■■ | 777777777460771 | 5 | 0,4 |  |  |  | 5 |  |  |  |
| 36 | NA | T1 *^1,00^ | ■■■■■■■■■■■■■■■■■■■■□■■■■■■■■■■■□□□□■■□■■■■ | 777777677760671 | 5 | 0,4 |  |  |  | 5 |  |  |  |
| 37 | 37 | T3 | ■■■■■■■■■■■■□■■■■■■■■■■■■■■■■■■■□□□□■■■■■■■ | 777737777760771 | 4 | 0,3 | 3 | 1 |  |  |  |  |  |
| 38 | 58 | T5_MAD2 | ■■■■■■■■■■■■■■■■■■■□■■□■■■■■■■■■□□□□■■■■■■■ | 777777557760771 | 4 | 0,3 | 4 |  |  |  |  |  |  |
| 39 | 60 | LAM4 | ■■■■■■■■■■■■■■■■■■■■□□□□■■■■■■■■□□□□■■■□■■■ | 777777607760731 | 4 | 0,3 | 2 |  |  |  | 2 |  |  |
| 40 | 91 | X3 | ■■■□□□□□□□□□□■■■■□■■■■■■■■■■■■■■□□□□■■■■■■■ | 700036777760771 | 4 | 0,3 | 2 | 2 |  |  |  |  |  |
| 41 | 177 | LAM9 | □■■■■■■■■■■■■■■■■■■■□□□□■■■■■■■■□□□□■■■■■■■ | 377777607760771 | 4 | 0,3 | 1 |  |  | 3 |  |  |  |
| 42 | 389 | LAM1 | ■■□■■■■■■■■■■■□■■■■■□□□□■■■■■■■■□□□□■■■■■■■ | 677767607760771 | 4 | 0,3 | 3 | 1 |  |  |  |  |  |
| 43 | 727 | Haarlem1 | ■■■■■■■■■■■■□■■■■■■■■■■■■□□□□□□■□□□□■■■□■■■ | 777737774020731 | 4 | 0,3 | 4 |  |  |  |  |  |  |
| 44 | 1367 | LAM5 | □■■■■■■■■■■■□■■■■■■■□□□□■■■■■■■■□□□□■■■■■■■ | 377737607760771 | 4 | 0,3 | 4 |  |  |  |  |  |  |
| 45 | 1661 | LAM2 | ■■□■■■■■■■■■□■■■■■■■□□□□■■■■■■■■□□□□■■□■■■■ | 677737607760671 | 4 | 0,3 | 2 |  |  | 2 |  |  |  |
| 46 | NA | *M. bovis* *^1,00^ | □□□□□□□□□□□□□□□□□□■■■■■■■■■■■■■■■■■□□□□□□□□ | 000000777776000 | 4 | 0,3 | 4 |  |  |  |  |  |  |
| 47 | NA | LAM9 *^1,00^ | ■■■□□■■■■■■■□■■■■■■■□□□□■■■■■■■■□□□□■■■■■■■ | 717737607760771 | 4 | 0,3 |  | 4 |  |  |  |  |  |
| 48 | NA | LAM9 *^1,00^ | ■■■■■■■■■■■■□■■■■□■■□□□□■■■■■■■■□□□□■■■■■■■ | 777736607760771 | 4 | 0,3 |  | 3 |  |  | 1 |  |  |
| 49 | NA | LAM9 *^1,00^ | ■■■■■■■■■■■■□■■■■■■■□□□□■■■■■■■■□□□□■■□■■■■ | 777737607760671 | 4 | 0,3 | 3 |  |  | 1 |  |  |  |
| 50 | 35 | Haarlem4 | ■■■■■■■■■■■■□■■■■■■■■■■■■■■■□□□■□□□□■■■■■■■ | 777737777420771 | 3 | 0,2 | 3 |  |  |  |  |  |  |
| 51 | 44 | T5 | ■■■■■■■■■■■■■■■■■■■■■■□■■■■■■■■■□□□□■■■■■■■ | 777777757760771 | 3 | 0,2 | 2 | 1 |  |  |  |  |  |
| 52 | 395 | T1 | ■■■■■■■■■■■■■■■■■■■■■■■■■■■■■■■■□□□□■□□□□□□ | 777777777760400 | 3 | 0,2 | 1 | 2 |  |  |  |  |  |
| 53 | 603 | Haarlem3 | ■■■■■■■■■■■■■■■■■■■■■■■■■■■□■■□■□□□□■■■□■■■ | 777777777320731 | 3 | 0,2 | 3 |  |  |  |  |  |  |
| 54 | 784 | T2-S | ■■■■■■■■□□■■■■■■■■■■■■■■■■■■■■■■□□□□■■■□■■■ | 776377777760731 | 3 | 0,2 | 2 |  |  |  | 1 |  |  |
| 55 | 821 | U | ■■□■■■■□□□□□□□□□□□□□□□□□□□□□□□□□□□□□□□■■■■■ | 674000000000171 | 3 | 0,2 | 2 |  |  |  |  |  | 1 |
| 56 | 863 | U | □□□□□□□□□□□□□□□□□□□□□□□□■■■■□□□□□□□□□□□□□□□ | 000000007400000 | 3 | 0,2 |  | 3 |  |  |  |  |  |
| 57 | 908 | LAM2 | ■■□■■■■■■■■■□■■■■■■■□□□□■■■■■■■■□□□□■■■□□□□ | 677737607760700 | 3 | 0,2 | 2 |  |  |  |  | 1 |  |
| 58 | 1698 | U | ■■■■■■■■■■■■□■□■■■■■□□□□■■■■■■□□□□□□□□□□■■■ | 777727607700031 | 3 | 0,2 |  | 3 |  |  |  |  |  |
| 59 | NA | T3 *^0,58^ | □□□□□□□□□□□□□□□□□□□□■■■■■■■■■■■■□□□□■□□□■■■ | 000000177760431 | 3 | 0,2 |  | 3 |  |  |  |  |  |
| 60 | NA | EAI1 *^0,88^ | ■■□□□□□□□□□□□□□■□□□□□□□■□□□□□□□□□□□□□□□□□□□ | 600004010000000 | 3 | 0,2 |  | 3 |  |  |  |  |  |
| 61 | NA | LAM10 *^0,94^ | ■■□□■■■■■■■■■□■■■■■■■□□□□■■■■■■□□□□□□■■■■■■ | 637757703740371 | 3 | 0,2 |  | 3 |  |  |  |  |  |
| 62 | NA | LAM2 *^0,82^ | ■■□■■□■■■■■■□■□□■■■■□□□□■■■■■■■■□□□□■■■■■■■ | 667723607760771 | 3 | 0,2 |  | 3 |  |  |  |  |  |
| 63 | NA | LAM2 *^0,82^ | ■■□■■■■■■■■■□□□■■■■■□□□□■■■■■■■■□□□□■■■■■■■ | 677707607760771 | 3 | 0,2 | 2 | 1 |  |  |  |  |  |
| 64 | NA | LAM2 *^0,81^ | ■■□■■■■■■■■■□■■■■■■■□□□□■■■■■■■■□□□□■■□□□□□ | 677737607760600 | 3 | 0,2 |  | 3 |  |  |  |  |  |
| 65 | NA | T3 *^0,95^ | ■■■□□□□□□□□□□□□□■■□■■■□□■■■■■■■■□□□□■□□■■□■ | 700003347760461 | 3 | 0,2 |  | 3 |  |  |  |  |  |
| 66 | NA | T3 *^1,00^ | ■■■■□■□□□□□□□□□□□□□□□□□□■■□■■■■■□□□□■■■■■■■ | 750000006760771 | 3 | 0,2 |  | 3 |  |  |  |  |  |
| 67 | 11 | EAI3_IND | ■□□■■■■■■■■■■■■■■■■■■■■■■■■■□□□□■□■■□□□■■■■ | 477777777413071 | 2 | 0,2 |  |  | 2 |  |  |  |  |
| 68 | 119 | X1 | ■■■■■■■■■■■■■■■■■□■■■■■■■■■■■■■■□□□□■■■■■■■ | 777776777760771 | 2 | 0,2 |  |  | 2 |  |  |  |  |
| 69 | 130 | LAM3 | ■■■■■■■■□□□■■■■■■■■■□□□□■■■■■■■■□□□□■■■□■■■ | 776177607760731 | 2 | 0,2 | 2 |  |  |  |  |  |  |
| 70 | 132 | U | ■■■■■■■■■■■■■■■■■■■■□□□□■■□□□□□□□□□□□□□□■■■ | 777777606000031 | 2 | 0,2 | 2 |  |  |  |  |  |  |
| 71 | 176 | LAM5 | ■■■■■■■■■■■■□■■■■■■■□□□□■■■■□■■■□□□□■■■■■■■ | 777737607560771 | 2 | 0,2 |  | 1 |  |  | 1 |  |  |
| 72 | 239 | U | ■■■■■■■■■■■■■■■■■■■■■■■■■■■■■■■■□□□□□□□□■■■ | 777777777760031 | 2 | 0,2 | 2 |  |  |  |  |  |  |
| 73 | 294 | Haarlem3 | ■□■■■■■■■■■■■■■■■■■■■■■■■■■■■■□■□□□□■■■■■■■ | 577777777720771 | 2 | 0,2 | 2 |  |  |  |  |  |  |
| 74 | 333 | LAM5 | ■■■■■■■■■■■■□■□■■■■■□□□□■■■■■■■■□□□□■■■■■■■ | 777727607760771 | 2 | 0,2 | 2 |  |  |  |  |  |  |
| 75 | 440 | LAM9 | ■■■■■■■■■■■□□□□■■■■■□□□□■■■■■■■■□□□□■■■■■■■ | 777607607760771 | 2 | 0,2 |  | 2 |  |  |  |  |  |
| 76 | 604 | LAM2 | ■■□■■■■■■■■■□■■■■■■■□□□□■■■■■■■■□□□□■■■■□■■ | 677737607760751 | 2 | 0,2 | 1 |  |  |  |  | 1 |  |
| 77 | 822 | LAM9 | ■■■■□■■■■■■■■■■■■■■■□□□□■■■■■■■■□□□□■■■■■■■ | 757777607760771 | 2 | 0,2 |  | 1 |  | 1 |  |  |  |
| 78 | 826 | LAM2 | ■■□■■■■■■■■■□■■■■■□■□□□□■■■■■■■■□□□□■■■■■■■ | 677737207760771 | 2 | 0,2 | 1 |  |  |  |  | 1 |  |
| 79 | 831 | S | ■■■■■■■■□□■■■■□■■■■■■■■■■■■■■■■■□□□□■■■■■■■ | 776367777760771 | 2 | 0,2 | 2 |  |  |  |  |  |  |
| 80 | 881 | U | ■■■■■■■■□□■■■■■■■■■■■■■■□□□□□□□□□□□□■■■□■■■ | 776377770000731 | 2 | 0,2 | 1 | 1 |  |  |  |  |  |
| 81 | 1926 | T1 | ■■■■■■■■■■■■■■■■■■■■■■■■■■■■■■■■□□□□■■■□□□■ | 777777777760701 | 2 | 0,2 | 2 |  |  |  |  |  |  |
| 82 | NA | LAM1 *^0,69^ | □□□□□□□■■■■■■■■■■■■■□□□□■■■■■■■■□□□□■■■■□□□ | 003777607760740 | 2 | 0,2 |  | 2 |  |  |  |  |  |
| 83 | NA | Haarlem3 *^0,77^ | □□□□■■■■■■■■■■■■■■■■■■■■■■■■■■□■□□□□■■■■■■■ | 037777777720771 | 2 | 0,2 |  | 2 |  |  |  |  |  |
| 84 | NA | LAM1 *^0,69^ | □□□■■■■□□□■■■■■■■■■■□□□□■■■■■■■■□□□□■■■□□□□ | 074377607760700 | 2 | 0,2 |  |  |  |  | 2 |  |  |
| 85 | NA | LAM5 *^0,97^ | ■□■■■■■■■□■□□■□■■■■■□□□□■■□□□■□□□□□□□□□□■■■ | 577227606100031 | 2 | 0,2 |  | 2 |  |  |  |  |  |
| 86 | NA | LAM5 *^0,98^ | ■□■■■■■■■■■■□■■■■■■■□□□□■■■■■■□□□□□□□□□□■■■ | 577737607700031 | 2 | 0,2 | 1 | 1 |  |  |  |  |  |
| 87 | NA | LAM9 *^1,00^ | ■□■■■■■■■■■■□■■■■■■■□□□□■■■■■■■■□□□□■■■■■■■ | 577737607760771 | 2 | 0,2 | 2 |  |  |  |  |  |  |
| 88 | NA | LAM2 *^0,82^ | ■■□■■■■■■■■□□■■■■■■■□□□□■■■■■■■■□□□□■■□■■■■ | 677637607760671 | 2 | 0,2 |  |  |  | 2 |  |  |  |
| 89 | NA | LAM2 *^0,82^ | ■■□■■■■■■■■□□■■■■■■■□□□□■■■■■■■■□□□□■■■■■■■ | 677637607760771 | 2 | 0,2 | 1 |  |  | 1 |  |  |  |
| 90 | NA | LAM1 *^0,66^ | ■■□■■■■■■■■■■■■■■■■■□□□□■■■□■■■■□□□□■■■■■■■ | 677777607360771 | 2 | 0,2 | 2 |  |  |  |  |  |  |
| 91 | NA | LAM9 *^1,00^ | ■■■■□■■■■■■■■■■■■■■■□□□□■■■■■■■■□□□□■■□■■■■ | 757777607760671 | 2 | 0,2 |  | 1 |  | 1 |  |  |  |
| 92 | NA | S *^0,78^ | ■■■■■■■■□□■■■□□■■■■■■■■■■■■■■■■■□□□□■■■■■■■ | 776347777760771 | 2 | 0,2 |  | 2 |  |  |  |  |  |
| 93 | NA | Haarlem3 ^0,77^ | ■■■■■■■■■■□□□■□■■■■■■■■■■■■■■■□■□□□□■■■■■■■ | 777427777720771 | 2 | 0,2 |  |  |  | 2 |  |  |  |
| 94 | NA | LAM9 ^1,00^ | ■■■■■■■■■■■□□■■■■■■■□□□□■■■■■■■■□□□□■■■■■■■ | 777637607760771 | 2 | 0,2 |  | 2 |  |  |  |  |  |
| 95 | NA | LAM9 ^0,99^ | ■■■■■■■■■■■■□■■■■■■■□□□□□■■■■■■■□□□□■■■■■■■ | 777737603760771 | 2 | 0,2 |  | 2 |  |  |  |  |  |
| 96 | NA | LAM9 ^1,00^ | ■■■■■■■■■■■■□■■■■■■■□□□□■□□■■■■■□□□□■■■■■■■ | 777737604760771 | 2 | 0,2 |  | 2 |  |  |  |  |  |
| 97 | NA | LAM9 ^0,97^ | ■■■■■■■■■■■■■■■■■■□□□□□□□■■■■■■■□□□□■■■■□□□ | 777777003760740 | 2 | 0,2 |  | 2 |  |  |  |  |  |
| 98 | NA | LAM9 ^1,00^ | ■■■■■■■■■■■■■■■■■■■■□□□□■■■■■■■■□□□□■□□■■■■ | 777777607760471 | 2 | 0,2 | 2 |  |  |  |  |  |  |
| 99 | NA | EAI1 ^0,56^ | ■■■■■■■■■■■■■■■■■■■■■■■■■■■□□□□□□□□□□□□□□□□ | 777777777000000 | 2 | 0,2 |  | 2 |  |  |  |  |  |
| 100 | 19 | EAI2_MANILLA | ■■□■■■■■■■■■■■■■■■■□□■■■■■■■□□□□■□■■■■■■■■■ | 677777477413771 | 1 | 0,1 |  |  | 1 |  |  |  |  |
| 101 | 49 | Haarlem3 | ■■■■■■■■■■■■■■■■■■■■■■■■■■■■■■□■□□□□■■■□■■■ | 777777777720731 | 1 | 0,1 | 1 |  |  |  |  |  |  |
| 102 | 52 | T2 | ■■■■■■■■■■■■■■■■■■■■■■■■■■■■■■■■□□□□■■■□■■■ | 777777777760731 | 1 | 0,1 | 1 |  |  |  |  |  |  |
| 103 | 71 | S | ■■■■■■■■□□■■□■■■■■■■■■■■■■■■■■■■□□□□■■■■■■■ | 776337777760771 | 1 | 0,1 |  |  |  | 1 |  |  |  |
| 104 | 81 | LAM9 | ■■■■■■■■■□■■■■■■■■■■□□□□■■■■■■■■□□□□■■■■■■■ | 777377607760771 | 1 | 0,1 |  | 1 |  |  |  |  |  |
| 105 | 92 | X3 | ■■■□□□□□□□□□■■■■■□■■■■■■■■■■■■■■□□□□■■■■■■■ | 700076777760771 | 1 | 0,1 |  | 1 |  |  |  |  |  |
| 106 | 95 | LAM6 | ■■■■■■■■■■■■■■■■■■■■□□□□■■■■□■■■□□□□■■■□■■■ | 777777607560731 | 1 | 0,1 |  | 1 |  |  |  |  |  |
| 107 | 121 | Haarlem3 | ■■■■■■■■■■■■■■■■■■■■■■■■■□■■■■□■□□□□■■■■■■■ | 777777775720771 | 1 | 0,1 | 1 |  |  |  |  |  |  |
| 108 | 134 | Haarlem3 | ■■■■■■■■■■■■■■■■■■■■■■■■■■■■■■□■□□□□■■□□■■■ | 777777777720631 | 1 | 0,1 | 1 |  |  |  |  |  |  |
| 109 | 154 | T1 | ■■■■□■■■■■■■■■■■■■■■■■■■■■■■■■■■□□□□■■■■■■■ | 757777777760771 | 1 | 0,1 |  | 1 |  |  |  |  |  |
| 110 | 159 | T1 Var- Toscana | ■■■■■■■■■■■■■□□□□□□□□□□■■■■■■■■■□□□□■■■■■■■ | 777740017760771 | 1 | 0,1 |  | 1 |  |  |  |  |  |
| 111 | 173 | T1 | ■■■■■■■■■■■■■■□■■■■■□■■■■■■■■■■■□□□□■■■■■■■ | 777767677760771 | 1 | 0,1 | 1 |  |  |  |  |  |  |
| 112 | 178 | LAM4 | ■□□■■■■■■■■■□■■■■■■■□□□□■■■■■■■■□□□□■■■□■■■ | 477737607760731 | 1 | 0,1 | 1 |  |  |  |  |  |  |
| 113 | 191 | T1 | □□■■■■■■■■■■■■■■■■■■■■■■■■■■■■■■□□□□■■■■■■■ | 177777777760771 | 1 | 0,1 | 1 |  |  |  |  |  |  |
| 114 | 205 | T1 | ■■■□■■■■■■■■■■■■■■■■■■■■■■■■■■■■□□□□■■■■■■■ | 737777777760771 | 1 | 0,1 | 1 |  |  |  |  |  |  |
| 115 | 211 | LAM3 | ■■■■■■■■□□□■□■■■■■■■□□□□■■■■■■■■□□□□■■■■■■■ | 776137607760771 | 1 | 0,1 | 1 |  |  |  |  |  |  |
| 116 | 216 | LAM9 | ■■■■■■■■■■■■□□■■■■■■□□□□■■■■■■■■□□□□■■■■■■■ | 777717607760771 | 1 | 0,1 |  |  |  |  | 1 |  |  |
| 117 | 218 | Haarlem1 | ■■■■■■■■■■■■□■■■■■■■■■■■■□□□□□□■□□□□■■■■■■■ | 777737774020771 | 1 | 0,1 | 1 |  |  |  |  |  |  |
| 118 | 237 | U- Haarlem3 | ■■■■■■■■■■■■■■■■■■■■■■■■■■■■■■□□□□□□□□□□□□□ | 777777777700000 | 1 | 0,1 |  | 1 |  |  |  |  |  |
| 119 | 290 | LAM8 | ■■■■■■■■■■■■■■■■■■■■□□□□■■□■■■■■□□□□■■■■■■■ | 777777606760771 | 1 | 0,1 | 1 |  |  |  |  |  |  |
| 120 | 336 | X1 | ■■■■■■■■■■■■■■■■■□■■■■■■■■■■■■■■□□□□■■■□■■■ | 777776777760731 | 1 | 0,1 |  | 1 |  |  |  |  |  |
| 121 | 373 | T1 | ■■■■■■■■■■■■■■■■■■■■■■■□■■■■■■■■□□□□■■■■■■■ | 777777767760771 | 1 | 0,1 | 1 |  |  |  |  |  |  |
| 122 | 377 | LAM2 | ■■□■□■■■■■■■□■■■■■■■□□□□■■■■■■■■□□□□■■■■■■■ | 657737607760771 | 1 | 0,1 | 1 |  |  |  |  |  |  |
| 123 | 378 | T1 | ■■■■■■■■■■■■■■■■■■■■□■■□■■■■■■■■□□□□■■■■■■■ | 777777667760771 | 1 | 0,1 | 1 |  |  |  |  |  |  |
| 124 | 379 | T1 | ■■■■■■■■■■■■■□□□□■□□□□□■■■■■■■■■□□□□■■■■■■■ | 777741017760771 | 1 | 0,1 |  |  |  | 1 |  |  |  |
| 125 | 390 | Haarlem3 | ■■■■■■■■■■■■■■■■■■■■■■■■■■■■■□□■□□□□■■■■■■■ | 777777777620771 | 1 | 0,1 |  |  |  |  | 1 |  |  |
| 126 | 435 | LAM9 | ■■■■■□□■■■■■■■■■■■■■□□□□■■■■■■■■□□□□■■■■■■■ | 763777607760771 | 1 | 0,1 | 1 |  |  |  |  |  |  |
| 127 | 478 | X2 | ■■□□□■■■■■■■■■■■■□■■■■■■■■■■■■■■□□□□■■□□□□■ | 617776777760601 | 1 | 0,1 |  | 1 |  |  |  |  |  |
| 128 | 509 | LAM9 | ■■■■■■■■■■■■■■■■■■■■□□□□■□■■■■■■□□□□■■■■■■■ | 777777605760771 | 1 | 0,1 | 1 |  |  |  |  |  |  |
| 129 | 522 | T1 | ■■■■■■■■■■■■■■■■■■■■■■■■■■■■■■■■□□□□■■■■■■□ | 777777777760770 | 1 | 0,1 | 1 |  |  |  |  |  |  |
| 130 | 610 | Haarlem1 | ■■■■■■■■■■■■■■□■■■■■■■■■■□□□□□□■□□□□■■■□■■■ | 777767774020731 | 1 | 0,1 |  | 1 |  |  |  |  |  |
| 131 | 725 | LAM5 | ■■■■■■■■■■■■□■■■■■■■□□□□■■■■■■■■□□□□■■■■■■□ | 777737607760770 | 1 | 0,1 |  |  | 1 |  |  |  |  |
| 132 | 749 | U | ■■■■■■■■■■■■■■■■■■■■□■■■■■■■■■■■■■■■■■■■■■■ | 777777677777771 | 1 | 0,1 |  |  |  |  | 1 |  |  |
| 133 | 774 | T1 | ■■■■■■■■■■■■■■■■■■■■■■■■□■■■■■■■□□□□■■■■■■■ | 777777773760771 | 1 | 0,1 | 1 |  |  |  |  |  |  |
| 134 | 879 | T1 | ■■■■■■■■■■■■■■□■■■■■■■■■■■■■■■■■□□□□■■□■■■■ | 777767777760671 | 1 | 0,1 | 1 |  |  |  |  |  |  |
| 135 | 880 | T1 | ■■■■■■■■■■■■■■■■■■□□□□□□□□□■■■■■□□□□■■■■■■■ | 777777000760771 | 1 | 0,1 |  |  |  |  | 1 |  |  |
| 136 | 888 | T1 | ■■■■■■■■■■■■■■■■■■■■■■■■■■■■■■■■□□□□■■□□■■■ | 777777777760631 | 1 | 0,1 | 1 |  |  |  |  |  |  |
| 137 | 914 | Haarlem3-S | ■■■■■■■■□□■■■■■■■■■■■■■■■■■■■■□■□□□□■■■■■■■ | 776377777720771 | 1 | 0,1 | 1 |  |  |  |  |  |  |
| 138 | 1053 | T1 | ■■■■■■■■■■■■■■□■■■■■□□■■■■■■■■■■□□□□■■■■■■■ | 777767637760771 | 1 | 0,1 |  | 1 |  |  |  |  |  |
| 139 | 1104 | T5 | ■■■■■■■■■■■■■■■■■■■■■■□■■■■■■□■■□□□□■■■■■■■ | 777777757660771 | 1 | 0,1 | 1 |  |  |  |  |  |  |
| 140 | 1147 | T1 | ■■■■■■■■■■■■■■■■■■■■□□□□□□□■□■■■□□□□■■■■■■■ | 777777600560771 | 1 | 0,1 | 1 |  |  |  |  |  |  |
| 141 | 1154 | LAM9 | ■■■■■■■■■■■■■■■■■■■■□□□□■■■■■■■■□□□□■■■■□■■ | 777777607760751 | 1 | 0,1 |  | 1 |  |  |  |  |  |
| 142 | 1209 | T1 | □□□□□□□□□□□□□□□□□□□■■■■■■■■■■■■■□□□□■■■■■■■ | 000000377760771 | 1 | 0,1 |  | 1 |  |  |  |  |  |
| 143 | 1225 | S | ■■■■■■■■□□■■■■■■■■■■■□□□□□□□□■■■□□□□■■■■■■■ | 776377700160771 | 1 | 0,1 |  | 1 |  |  |  |  |  |
| 144 | 1271 | S | ■■■■■■■■□□■■■■■■■■■■□■■■■■■■■■■■□□□□■■■■■■■ | 776377677760771 | 1 | 0,1 | 1 |  |  |  |  |  |  |
| 145 | 1321 | LAM1-LAM4 | ■■□■■■■■■■■■■■■■■■■■□□□□■■■■■■■■□□□□■■■□■■■ | 677777607760731 | 1 | 0,1 | 1 |  |  |  |  |  |  |
| 146 | 1328 | Haarlem1 | ■■■■■■■■■■■■■■■■■■□□□□■■■□□□□□□■□□□□■■■■■■■ | 777777034020771 | 1 | 0,1 |  | 1 |  |  |  |  |  |
| 147 | 1471 | LAM11_ZWE | ■■■■■■■■■■■■□■■■■■■■□□□□■■□□□□■■□□□□■■■■■■■ | 777737606060771 | 1 | 0,1 | 1 |  |  |  |  |  |  |
| 148 | 1530 | LAM9 | ■■■■■■■■■■■■■■■■■■■■□□□□■■■■■■■■□□□□■■■□□■■ | 777777607760711 | 1 | 0,1 |  |  |  | 1 |  |  |  |
| 149 | 1552 | Haarlem1 | ■■■■■■■■■■■■■■■■■■■■■■■■■□□□□□□■□□□□■■□□■■■ | 777777774020631 | 1 | 0,1 |  |  |  | 1 |  |  |  |
| 150 | 1561 | U | ■■■■■■■■■■■■■■■■■■■■■■■■■□□□□□□■□□□□□■■■■■■ | 777777774020371 | 1 | 0,1 | 1 |  |  | 1 |  |  |  |
| 151 | 1630 | T1 | ■■■■■■■■■■■■■■■■■■■■□□□□□□■■■■■■□□□□■■■■■■■ | 777777601760771 | 1 | 0,1 | 1 |  |  |  |  |  |  |
| 152 | 1693 | LAM5 | ■■■□■■■■■■■■□■■■■■■■□□□□■■■■■■■■□□□□■■■■■■■ | 737737607760771 | 1 | 0,1 | 1 |  |  |  |  |  |  |
| 153 | 1695 | LAM2 | ■■□■■■■■■■■■□■■■■■■■□□□□■■■■■■■■□□□□■■■■■■□ | 677737607760770 | 1 | 0,1 |  |  |  | 1 |  |  |  |
| 154 | 1700 | T1 | ■■■■□■□■■■■■■■■■■■■■■■■■■■□■■■■■□□□□■■■■■■■ | 753777776760771 | 1 | 0,1 |  | 1 |  |  |  |  |  |
| 155 | 1711 | LAM2 | ■■□■■■■■■□■■□■■■■■■■□□□□■■■■■■■■□□□□■■■■■■■ | 677337607760771 | 1 | 0,1 |  |  |  | 1 |  |  |  |
| 156 | 1718 | X1-LAM9 | ■■□■■■■■■■■■■■■■■□■■□□□□■■■■■■■■□□□□■■■■■■■ | 677776607760771 | 1 | 0,1 |  |  | 1 |  |  |  |  |
| 157 | 1751 | X3 | ■■■□□□□□□□□□■■□■■□■■■■■■■■■■■■■■□□□□■■■■■■■ | 700066777760771 | 1 | 0,1 |  | 1 |  |  |  |  |  |
| 158 | 1815 | LAM1-LAM8 | ■■□■■■■■■■■■■■■■■■■■□□□□■■□■■■■■□□□□■■■■■■■ | 677777606760771 | 1 | 0,1 | 1 |  |  |  |  |  |  |
| 159 | 1933 | LAM9 | ■■■■■■■■■■■■■■■■■■■■□□□□■■■■■■■■□□□□■■■■■■□ | 777777607760770 | 1 | 0,1 |  |  |  | 1 |  |  |  |
| 160 | NA | Familia36 *^1,00^ | □□□□□□□□□□□□□□□□□□□□□□□□□□■■■□□□□□□□□□□□□□□ | 000000001600000 | 1 | 0,1 |  |  | 1 |  |  |  |  |
| 161 | NA | Familia36 *^1,00^ | □□□□□□□□□□□□□□□□□□□□□□□□■■■■■□□□□□□□□■■■■■■ | 000000007600371 | 1 | 0,1 | 1 |  |  |  |  |  |  |
| 162 | NA | *M. bovis* *^1,00^ | □□□□□□□□□□□□□□□□□■■■■■■■■■■■■■■■■■■□□□□□□□□ | 000001777776000 | 1 | 0,1 |  |  | 1 |  |  |  |  |
| 163 | NA | *M africanum* *^0,99^ | □□□□□□□□□□□□□■■■■■■■■■■■■■■■■■■■■■■□□□□□□□□ | 000037777776000 | 1 | 0,1 | 1 |  |  |  |  |  |  |
| 164 | NA | Haarlem3 *^0,77^ | □□□□■■□□■□■□■■□■■■■■■■■■■■■□■■□■□□□□■■■■□□■ | 031267777320741 | 1 | 0,1 |  | 1 |  |  |  |  |  |
| 165 | NA | LAM2 *^0,76^ | □□□□■■■■■■■■□■□■■■■■□□□□■■■■□■■■□□□□■■■■■■■ | 037727607560771 | 1 | 0,1 |  | 1 |  |  |  |  |  |
| 166 | NA | LAM2 *^0,81^ | □□□■■■■□□□■■□■■■■■■■□□□□■■■■■■■■□□□□■■■□□□□ | 074337607760700 | 1 | 0,1 |  | 1 |  |  |  |  |  |
| 167 | NA | LAM2 *^0,81^ | □□□■■■■■■□■■□■□■■■■■□□□□■■■■■■■■□□□□■■■■■■■ | 077327607760771 | 1 | 0,1 |  |  | 1 |  |  |  |  |
| 168 | NA | LAM2 *^0,81^ | □□□■■■■■■■■■□■□■■■■■□□□□■■■■■■■■□□□□■■□■■■■ | 077727607760671 | 1 | 0,1 | 1 |  |  |  |  |  |  |
| 169 | NA | LAM9 *^0,99^ | □□■□□□□□■□□□□□□■■■□□□□□□□■□□□■■□□□□□□□□■□□■ | 101007002140041 | 1 | 0,1 | 1 |  |  |  |  |  |  |
| 170 | NA | Haarlem1 *^1,00^ | □□■□□■■□■□□□■■□■■■□□□□■■■□□□□□□□□□□□■□□■□□■ | 115067034000441 | 1 | 0,1 |  | 1 |  |  |  |  |  |
| 171 | NA | LAM 9 *^1,00^ | □□■□■■■■■■■■□■□■■■■■□□□□■■■■■■■■□□□□■■■■■■■ | 137727607760771 | 1 | 0,1 |  | 1 |  |  |  |  |  |
| 172 | NA | LAM9 *^1,00^ | □□■■■■□■■□■■■■□■■■■■□□□□■■□□□■■■□□□□■■■■■□■ | 173367606160761 | 1 | 0,1 |  | 1 |  |  |  |  |  |
| 173 | NA | S *^0,78^ | □□■■■■■■□□■■■■■■■■■■■■■■■■■■■■■■□□□□■■■■■■■ | 176377777760771 | 1 | 0,1 |  | 1 |  |  |  |  |  |
| 174 | NA | LAM9 *^1,00^ | □□■■■■■■■□■■□■■■■■■■□□□□■■■■■■■■□□□□■■■■■■■ | 177337607760771 | 1 | 0,1 | 1 |  |  |  |  |  |  |
| 175 | NA | LAM9 *^1,00^ | □□■■■■■■■■■■□■□■■■■■□□□□■■■■■■■■□□□□■■□■■■■ | 177727607760671 | 1 | 0,1 | 1 |  |  |  |  |  |  |
| 176 | NA | Familia33 *^1,00^ | □□■■■■■■■■■■■■□■■■■■■■■■■■■■■■■■□■■■■■□■■■■ | 177767777767671 | 1 | 0,1 | 1 |  |  |  |  |  |  |
| 177 | NA | LAM9 *^1,00^ | □□■■■■■■■■■■■■■■■■■■□□□□■■■■■■■■□□□□■■□■■■■ | 177777607760671 | 1 | 0,1 |  | 1 |  |  |  |  |  |
| 178 | NA | LAM9 *^1,00^ | □□■■■■■■■■■■■■■■■■■■□□□□■■■■■■■■□□□□■■■■■■■ | 177777607760771 | 1 | 0,1 | 1 |  |  |  |  |  |  |
| 179 | NA | EAI1 *^1,00^ | □■■■□□□□■■□□□□■■■■□□□□□□□□□□□□□□□□□□□□□□□■□ | 341417000000010 | 1 | 0,1 |  |  | 1 |  |  |  |  |
| 180 | NA | LAM7 *^1,00^ | □■■■■■■■□□□■■■■■■■■□□□□□□□□□□□□□□□□□□□■■■■■ | 376177400000171 | 1 | 0,1 |  |  | 1 |  |  |  |  |
| 181 | NA | T1 *^1,00^ | □■■■■■■■■□■□■■□■■■■■■■■■■■■□□■■■□□□□■■□□■■■ | 377267777160631 | 1 | 0,1 |  | 1 |  |  |  |  |  |
| 182 | NA | LAM9 *^1,00^ | □■■■■■■■■■■■□■■■■■■■□□□□■■□□□■■■□□□□■■■■■■■ | 377737606160771 | 1 | 0,1 | 1 |  |  |  |  |  |  |
| 183 | NA | T1 *^1,00^ | □■■■■■■■■■■■■■■■■■■■■■■□■■■■■■■■□□□□■■■■■■■ | 377777767760771 | 1 | 0,1 | 1 |  |  |  |  |  |  |
| 184 | NA | T2 *^0,98^ | □■■■■■■■■■■■■■■■■■■■■■■■■■■■■■■■□□□□■■■□□□□ | 377777777760700 | 1 | 0,1 | 1 |  |  |  |  |  |  |
| 185 | NA | LAM2 *^0,81^ | ■□□□■■■■■■■■□■■■■■■■□□□□■■■■■■■■□□□□■■□□■■■ | 437737607760631 | 1 | 0,1 |  | 1 |  |  |  |  |  |
| 186 | NA | LAM9 *^1,00^ | ■□■■■□■■■■■■■□■■■■■■□□□□■■■■□■■■□□□□■■■■■■■ | 567757607560771 | 1 | 0,1 |  | 1 |  |  |  |  |  |
| 187 | NA | T1 *^1,00^ | ■□■■■■■■■■■■■■□■■■■■■■■■■■□□■■■■□□□□□■□■■■□ | 577767776360270 | 1 | 0,1 | 1 |  |  |  |  |  |  |
| 188 | NA | EAI1 *^1,00^ | ■□■■■■■■■■■■■■■■■■■■□□□□■■□□□□□□□□□□□□□□■■■ | 577777606000031 | 1 | 0,1 | 1 |  |  |  |  |  |  |
| 189 | NA | Familia36 *^1,00^ | ■■□□□□□□□□□□□□□□□□□□□□□□□□□□■■■□□□□□□□□□□□□ | 600000000340000 | 1 | 0,1 |  |  | 1 |  |  |  |  |
| 190 | NA | Familia36 *^1,00^ | ■■□□□□□□□□□□□□□□□□□□□□□□■□□□■■□□□□□□■■■□□□□ | 600000004300700 | 1 | 0,1 |  |  | 1 |  |  |  |  |
| 191 | NA | S *^0,78^ | ■■□□□□□□□□■■■■■■■■■■■■■■■■■■■■■■□□□□■■■■■■■ | 600377777760771 | 1 | 0,1 | 1 |  |  |  |  |  |  |
| 192 | NA | T1 *^1,00^ | ■■□□□□□□□■■■■■■■■■■■■■■■■■■■■■■■□□□□■■□■■■■ | 600777777760671 | 1 | 0,1 | 1 |  |  |  |  |  |  |
| 193 | NA | LAM1 *^0,51^ | ■■□□□□■■■■■■■■■■■■■■□□□□■■■■□■■■□□□□■■■■■■■ | 607777607560771 | 1 | 0,1 |  |  |  | 1 |  |  |  |
| 194 | NA | T4 *^1,00^ | ■■□□□■■■■■■■■■□□□□□□□□□□■■■■■■■■□□□□■■■■■■■ | 617760007760771 | 1 | 0,1 | 1 |  |  |  |  |  |  |
| 195 | NA | LAM2 *^0,82^ | ■■□■■■■■■■□■□□□■■■■■□□□□■■■■■■■■□□□□■■■■□■■ | 677507607760751 | 1 | 0,1 | 1 |  |  |  |  |  |  |
| 196 | NA | LAM2 *^0,82^ | ■■□■■■■■■■■■□■□■■■■■□□□□■■□■■■■■□□□□■■□□■■■ | 677727606760631 | 1 | 0,1 |  | 1 |  |  |  |  |  |
| 197 | NA | LAM2 *^0,77^ | ■■□■■■■■■■■■□■□■■■■■□□□□■■■■□■■■□□□□■■■■■■■ | 677727607560771 | 1 | 0,1 |  | 1 |  |  |  |  |  |
| 198 | NA | LAM2 *^0,82^ | ■■□■■■■■■■■■□■□■■■■■□□□□■■■■■□□■□□□□■■■■■□□ | 677727607620760 | 1 | 0,1 |  | 1 |  |  |  |  |  |
| 199 | NA | LAM5 *^0,81^ | ■■□■■■■■■■■■□■□■■■■■□□□□■■■■■■□□□□□□■■■■■■■ | 677727607700771 | 1 | 0,1 | 1 |  |  |  |  |  |  |
| 200 | NA | LAM2 *^0,82^ | ■■□■■■■■■■■■□■■□■■■■□□□□■■■■■■■■□□□□■■■■■■■ | 677733607760771 | 1 | 0,1 |  | 1 |  |  |  |  |  |
| 201 | NA | LAM2 *^0,82^ | ■■□■■■■■■■■■□■■■□■■■□□□□■■■■■■■■□□□□■■■■■□□ | 677735607760760 | 1 | 0,1 | 1 |  |  |  |  |  |  |
| 202 | NA | Familia34 *^0,94^ | ■■□■■■■■■■■■□■■■■■■■□□□□□□□□□□□□□□□□□□□□□□□ | 677737600000000 | 1 | 0,1 | 1 |  |  |  |  |  |  |
| 203 | NA | LAM2 *^0,82^ | ■■□■■■■■■■■■□■■■■■■■□□□□□■■■■■■■□□□□■■■■■■■ | 677737603760771 | 1 | 0,1 |  |  |  | 1 |  |  |  |
| 204 | NA | LAM2 *^0,82^ | ■■□■■■■■■■■■□■■■■■■■□□□□■■□□■■■■□□□□■■■■■■■ | 677737606360771 | 1 | 0,1 |  | 1 |  |  |  |  |  |
| 205 | NA | LAM2 *^0,82^ | ■■□■■■■■■■■■□■■■■■■■□□□□■■□■■■■■□□□□■■■■■■■ | 677737606760771 | 1 | 0,1 |  | 1 |  |  |  |  |  |
| 206 | NA | EAI1 *^1,00^ | ■■□■■■■■■■■■□■■■■■■■□□□□■■■□□□□□□□□□□□□□□□□ | 677737607000000 | 1 | 0,1 |  | 1 |  |  |  |  |  |
| 207 | NA | LAM2 *^0,77^ | ■■□■■■■■■■■■□■■■■■■■□□□□■■■■□□□■□□□□■■■■□□□ | 677737607420740 | 1 | 0,1 |  | 1 |  |  |  |  |  |
| 208 | NA | LAM5 *^0,80^ | ■■□■■■■■■■■■□■■■■■■■□□□□■■■■□■□□□□□□■■■■■■■ | 677737607500771 | 1 | 0,1 |  |  |  |  | 1 |  |  |
| 209 | NA | LAM2 *^0,82^ | ■■□■■■■■■■■■□■■■■■■■□□□□■■■■■□□■□□□□■■■■■□■ | 677737607620761 | 1 | 0,1 |  |  |  | 1 |  |  |  |
| 210 | NA | LAM2 *^0,82^ | ■■□■■■■■■■■■□■■■■■■■□□□□■■■■■■■■□□□□□■■■■■■ | 677737607760371 | 1 | 0,1 | 1 |  |  |  |  |  |  |
| 211 | NA | LAM2 *^0,82^ | ■■□■■■■■■■■■□■■■■■■■□□□□■■■■■■■■□□□□■□■■■■■ | 677737607760571 | 1 | 0,1 | 1 |  |  |  |  |  |  |
| 212 | NA | LAM2 *^0,82^ | ■■□■■■■■■■■■□■■■■■■■□□□□■■■■■■■■□□□□■■□□□□■ | 677737607760601 | 1 | 0,1 | 1 |  |  |  |  |  |  |
| 213 | NA | LAM1 *^0,66^ | ■■□■■■■■■■■■■□□■■■■■□□□□■■■■■■■■□□□□■■□■■■■ | 677747607760671 | 1 | 0,1 |  | 1 |  |  |  |  |  |
| 214 | NA | LAM1 *^0,66^ | ■■□■■■■■■■■■■■□■■■■■□□□□□□□■■■■■□□□□■■■■■■■ | 677767600760771 | 1 | 0,1 |  | 1 |  |  |  |  |  |
| 215 | NA | T1 *^0,92^ | ■■□■■■■■■■■■■■■■■■■□□□□■■■■■■■■□□□□■■■■■■■■ | 677777417741771 | 1 | 0,1 |  | 1 |  |  |  |  |  |
| 216 | NA | LAM1 *^0,66^ | ■■□■■■■■■■■■■■■■■■■■□□□□■■■■■■■■□□□□□■■■■■■ | 677777607760371 | 1 | 0,1 | 1 |  |  |  |  |  |  |
| 217 | NA | T1 *^1,00^ | ■■□■■■■■■■■■■■■■■■■■■■□■■■■■■□■■□□□□■■■■■■■ | 677777757660771 | 1 | 0,1 |  | 1 |  |  |  |  |  |
| 218 | NA | S *^0,83^ | ■■■□□□□■□□■□■■□■■□■■■■■■■■■■■■■■□□□□■■■■■■■ | 702266777760771 | 1 | 0,1 | 1 |  |  |  |  |  |  |
| 219 | NA | T3 *^1,00^ | ■■■□□□■□□□□□□□□□■□□■■■□□■■■■■■■■□□□□■□□■■■■ | 704002347760471 | 1 | 0,1 |  | 1 |  |  |  |  |  |
| 220 | NA | LAM9 *^1,00^ | ■■■□□□■■■■□■□■■■■■■■□□□□■■■■■■■■□□□□■■■■■■■ | 707537607760771 | 1 | 0,1 |  | 1 |  |  |  |  |  |
| 221 | NA | LAM9 *^1,00^ | ■■■□□□■■■■■■□■□■■■■■□□□□■■■■■■■■□□□□■■■□■■■ | 707727607760731 | 1 | 0,1 |  | 1 |  |  |  |  |  |
| 222 | NA | LAM9 *^1,00^ | ■■■□□□■■■■■■□■□■■■■■□□□□■■■■■■■■□□□□■■■■■■■ | 707727607760771 | 1 | 0,1 |  | 1 |  |  |  |  |  |
| 223 | NA | LAM9 *^1,00^ | ■■■□□□■■■■■■□■■■■■■■□□□□■■■■■■■■□□□□■■□■■□■ | 707737607760661 | 1 | 0,1 | 1 |  |  |  |  |  |  |
| 224 | NA | LAM9 *^1,00^ | ■■■□□■■■■■■■□■■■■■■■□□□□■■■■■■■■□□□□■■■□■■■ | 717737607760731 | 1 | 0,1 | 1 |  |  |  |  |  |  |
| 225 | NA | LAM9 *^1,00^ | ■■■□■■■■■■■■□■■■■■■■□□□□■■■■■■■■□□□□■■■□□■■ | 737737607760711 | 1 | 0,1 | 1 |  |  |  |  |  |  |
| 226 | NA | T1 *^1,00^ | ■■■□■■■■■■■■■■■■■■■□■■□■■■■■■■■■□□□□■■■■■■■ | 737777557760771 | 1 | 0,1 |  | 1 |  |  |  |  |  |
| 227 | NA | S *^0,78^ | ■■■■□□■■□□■■■■■■■■■■■■■□■■■■■■■■□□□□■■■■■■■ | 746377767760771 | 1 | 0,1 |  | 1 |  |  |  |  |  |
| 228 | NA | T1 *^1,00^ | ■■■■□■□■■■■□■■■■■■■■■■■■■■□■■■■■□□□□■■■■■■■ | 753677776760771 | 1 | 0,1 |  |  |  |  |  | 1 |  |
| 229 | NA | T1 *^0,99^ | ■■■■□■□■■■■■■■■■■■■■■■■■■■□■■■■■□□□□■■■□□■■ | 753777776760711 | 1 | 0,1 | 1 |  |  |  |  |  |  |
| 230 | NA | LAM *^1,00^ | ■■■■□■■■■■■■□■■■■■■■□□□□■■■■■■■■□□□□■■■■■■■ | 757737607760771 | 1 | 0,1 | 1 |  |  |  |  |  |  |
| 231 | NA | LAM9 *^1,00^ | ■■■■□■■■■■■■■■□□■■■■□□□□■■■■■■■■□□□□■■■■■■■ | 757763607760771 | 1 | 0,1 |  | 1 |  |  |  |  |  |
| 232 | NA | LAM9 *^1,00^ | ■■■■□■■■■■■■■■□■■■■■□□□□■■■■■■■■□□□□■■■■■■■ | 757767607760771 | 1 | 0,1 |  | 1 |  |  |  |  |  |
| 233 | NA | LAM9 *^1,00^ | ■■■■■□□□■■■■■■■■■■■■□□□□■■■■■■■■□□□□■■■■■■■ | 761777607760771 | 1 | 0,1 | 1 |  |  |  |  |  |  |
| 234 | NA | S *^0,78^ | ■■■■■□■■□□■■■■■■■■■■■■■■■■■■□■■■□□□□■■■■■■■ | 766377777560771 | 1 | 0,1 |  | 1 |  |  |  |  |  |
| 235 | NA | LAM9 *^1,00^ | ■■■■■□■■■■■■□□□■■■■■□□□□■■■■■■■■□□□□■■■■■■■ | 767707607760771 | 1 | 0,1 |  | 1 |  |  |  |  |  |
| 236 | NA | LAM9 *^1,00^ | ■■■■■□■■■■■■■■□■■■■■□□□□■■■■■■■■□□□□■■■■■■■ | 767767607760771 | 1 | 0,1 |  | 1 |  |  |  |  |  |
| 237 | NA | T3*^1,00^ | ■■■■■■□□□□□□□□□□□□■■□■□□□■■■■■■■□□□□■■■■■■■ | 770000643760771 | 1 | 0,1 | 1 |  |  |  |  |  |  |
| 238 | NA | S *^0,98^ | ■■■■■■□□□□□□□□□■■■■■■■■■■■■■■□□□□□□□□■■■■■■ | 770007777600371 | 1 | 0,1 | 1 |  |  |  |  |  |  |
| 239 | NA | T2 *^0,98^ | ■■■■■■□□■□□■■■□■■■■■■■■■■■■■■■■■□□□□■■■□□□□ | 771167777760700 | 1 | 0,1 | 1 |  |  |  |  |  |  |
| 240 | NA | Haarlem1 *^1,00^ | ■■■■■■□■■□■■■■■■■■■■■■■■■□□□□□□■□□□□■■■■■■■ | 773377774020771 | 1 | 0,1 | 1 |  |  |  |  |  |  |
| 241 | NA | T1 *^1,00^ | ■■■■■■□■■□■■■■■■■■■■■■■■■■■■■□■■□□□□■■■■■■■ | 773377777660771 | 1 | 0,1 |  | 1 |  |  |  |  |  |
| 242 | NA | LAM9 *^1,00^ | ■■■■■■□■■■■■■■□■■■■■□□□□■■■■■■■■□□□□■■■■■■■ | 773767607760771 | 1 | 0,1 |  | 1 |  |  |  |  |  |
| 243 | NA | LAM9*^1,00^ | ■■■■■■■□□□■■□■■■■■■■□□□□■■■■■■■■□□□□■■■■■■■ | 774337607760771 | 1 | 0,1 | 1 |  |  |  |  |  |  |
| 244 | NA | LAM9 *^1,00^ | ■■■■■■■□■■■■□■■■■■■■□□□□■■■■■■■■□□□□■■■■■■■ | 775737607760771 | 1 | 0,1 |  | 1 |  |  |  |  |  |
| 245 | NA | LAM7 *^1,00^ | ■■■■■■■■□□□■□■■■■■■□□□□□□□□□□□□□□□□□□□■■■■■ | 776137400000171 | 1 | 0,1 |  | 1 |  |  |  |  |  |
| 246 | NA | LAM3 *^1,00^ | ■■■■■■■■□□□■■■■■■■■■□□□□■■■■■■■■□□□□■■■■■□□ | 776177607760760 | 1 | 0,1 | 1 |  |  |  |  |  |  |
| 247 | NA | LAM9 *^0,99^ | ■■■■■■■■□□■□□□■■■■■■□□□□■■■■■■■■□□□□■■■■■■■ | 776217607760771 | 1 | 0,1 |  |  |  | 1 |  |  |  |
| 248 | NA | S *^0,98^ | ■■■■■■■■□□■□□■■■■■■■■■■■■■■■■■■■□□□□■■■■■■■ | 776237777760771 | 1 | 0,1 |  | 1 |  |  |  |  |  |
| 249 | NA | LAM9 *^1,00^ | ■■■■■■■■□□■■□■□■■■■■□□□□■■■■■■■■□□□□■■■□■■■ | 776327607760731 | 1 | 0,1 | 1 |  |  |  |  |  |  |
| 250 | NA | S *^0,75^ | ■■■■■■■■□□■■□■□■■■■■■■■■■■■■■□□□□□□□□■■■■■■ | 776327777600371 | 1 | 0,1 |  | 1 |  |  |  |  |  |
| 251 | NA | S *^0,93^ | ■■■■■■■■□□■■□■■■■■■■■■■□■■■■■■■■□□□□■■■■■■■ | <<<<<<<<< | 1 | 0,1 | 1 |  |  |  |  |  |  |
| 252 | NA | S *^0,75^ | ■■■■■■■■□□■■□■■■■■■■■■■■■■■■■□□□□□□□□■■■■■■ | 776337777600371 | 1 | 0,1 | 1 |  |  |  |  |  |  |
| 253 | NA | S *^0,93^ | ■■■■■■■■□□■■□■■■■■■■■■■■■■■■■■■■□□□□■■■■□■■ | 776337777760751 | 1 | 0,1 | 1 |  |  |  |  |  |  |
| 254 | NA | LAM10 *^0,86^ | ■■■■■■■■□□■■■■□■■■■■■□□□□□□□□■■■□□□□■■■■■■■ | 776367700160771 | 1 | 0,1 |  | 1 |  |  |  |  |  |
| 255 | NA | S *^0,78^ | ■■■■■■■■□□■■■■□■■■■■■■■□■■■■■■■■□□□□■■■■■■■ | 776367767760771 | 1 | 0,1 |  |  |  |  |  |  |  |
| 256 | NA | S *^0,78^ | ■■■■■■■■□□■■■■□■■■■■■■■■■■■■■■■■□□□□■■□■■■□ | 776367777760670 | 1 | 0,1 |  | 1 |  |  |  |  |  |
| 257 | NA | Familia33 *^1,00^ | ■■■■■■■■■□□□■■■□■■■■■■■■■■■■■■■■■■■■■■□□□□□ | 777073777777600 | 1 | 0,1 | 1 |  |  |  |  |  |  |
| 258 | NA | LAM5 *^0,97^ | ■■■■■■■■■□■■□□□■■■■■□□□□■■□□□■□□□□□□■■□■■□■ | 777307606100661 | 1 | 0,1 |  | 1 |  |  |  |  |  |
| 259 | NA | LAM9 *^1,00^ | ■■■■■■■■■□■■□■□□□■■■□□□□■■■■■■■□□□□□□■□■■□□ | 777321607740260 | 1 | 0,1 |  | 1 |  |  |  |  |  |
| 260 | NA | LAM9 *^0,99^ | ■■■■■■■■■□■■■□□■■■□□□□□□■■□■■■■□□□□□■■□■■□■ | 777347006740661 | 1 | 0,1 |  | 1 |  |  |  |  |  |
| 261 | NA | LAM9 *^1,00^ | ■■■■■■■■■□■■■■□■□■□■□□□□■■■■■■■■□□□□■■□□■□□ | 777365207760620 | 1 | 0,1 |  | 1 |  |  |  |  |  |
| 262 | NA | LAM9 *^1,00^ | ■■■■■■■■■□■■■■■■■■■■□□□□■■□□■■□□□□□□■■□■■□■ | 777377606300661 | 1 | 0,1 |  | 1 |  |  |  |  |  |
| 263 | NA | Haarlem3*^0,77^ | ■■■■■■■■■■□□□□■■■■■■■■■■■■■■■■□■□□□□■■■■■■■ | 777417777720771 | 1 | 0,1 |  | 1 |  |  |  |  |  |
| 264 | NA | Haarlem1*^1,00^ | ■■■■■■■■■■□□□■■■■■■■□■■■■□□□□□□■□□□□■■■□■■■ | 777437674020731 | 1 | 0,1 | 1 |  |  |  |  |  |  |
| 265 | NA | LAM9 *^0,89^ | ■■■■■■■■■■■□■■■■■■■■□□□□■■■■■■■■□□□□■■□□□□□ | 777677607760600 | 1 | 0,1 | 1 |  |  |  |  |  |  |
| 266 | NA | T1*^1,00^ | ■■■■■■■■■■■□■■■■■■■■□■■■■■■■■■■■□□□□■■□■■■■ | 777677677760671 | 1 | 0,1 |  |  |  | 1 |  |  |  |
| 267 | NA | LAM9 *^1,00^ | ■■■■■■■■■■■■□□□■■■■■□□□□■■■■■■■■□□□□■■■■■■■ | 777707607760771 | 1 | 0,1 |  | 1 |  |  |  |  |  |
| 268 | NA | LAM *^0,99^ | ■■■■■■■■■■■■□□■■■■■■□□□□□■■■■■■■□□□□■■■■■■■ | 777717603760771 | 1 | 0,1 |  | 1 |  |  |  |  |  |
| 269 | NA | T1*^1,00^ | ■■■■■■■■■■■■□■□□■■■■■■■■■■■■□□■■□□□□■■□■■■■ | 777723777460671 | 1 | 0,1 |  | 1 |  |  |  |  |  |
| 270 | NA | T2 *^0,91^ | ■■■■■■■■■■■■□■□■■■■■□□□□■■■■■■■□□□□□□□□□□□□ | 777727607740000 | 1 | 0,1 | 1 |  |  |  |  |  |  |
| 271 | NA | T2 *^0,91^ | ■■■■■■■■■■■■□■□■■■■■□□■□■■■■■■□□□□□□□□□□□■■ | 777727627700011 | 1 | 0,1 | 1 |  |  |  |  |  |  |
| 272 | NA | LAM9 ^1,00^ | ■■■■■■■■■■■■□■■■□□■■□□□□■■■■■■■■□□□□■■■■■■■ | 777734607760771 | 1 | 0,1 |  | 1 |  |  |  |  |  |
| 273 | NA | Familia34 *^1,00^ | ■■■■■■■■■■■■□■■■■□■■□□□□□□□□□□□□□□□□□□□□□□□ | 777736600000000 | 1 | 0,1 | 1 |  |  |  |  |  |  |
| 274 | NA | LAM9 *^1,00^ | ■■■■■■■■■■■■□■■■■■■■□□□□■□□□□■■■□□□□■■■□■■■ | 777737604160731 | 1 | 0,1 |  | 1 |  |  |  |  |  |
| 275 | NA | LAM9 *^1,00^ | ■■■■■■■■■■■■□■■■■■■■□□□□■■■■■■□■□□□□■■■■■■■ | 777737607720771 | 1 | 0,1 |  | 1 |  |  |  |  |  |
| 276 | NA | LAM9 *^1,00^ | ■■■■■■■■■■■■□■■■■■■■□□□□■■■■■■■■□□□□■■□■□■■ | 777737607760651 | 1 | 0,1 | 1 |  |  |  |  |  |  |
| 277 | NA | LAM9 *^1,00^ | ■■■■■■■■■■■■□■■■■■■■□□□□■■■■■■■■□□□□■■□■■□□ | 777737607760660 | 1 | 0,1 |  | 1 |  |  |  |  |  |
| 278 | NA | T1*^1,00^ | ■■■■■■■■■■■■□■■■■■■■□■■■■■■■□■■■□□□□■■■■■■■ | 777737677560771 | 1 | 0,1 |  |  | 1 |  |  |  |  |
| 279 | NA | Haarlem1* ^1,00^ | ■■■■■■■■■■■■□■■■■■■■■■■□□□□□□□□■□□□□■■■■■■■ | 777737760020771 | 1 | 0,1 |  | 1 |  |  |  |  |  |
| 280 | NA | Haarlem1*^1,00^ | ■■■■■■■■■■■■□■■■■■■■■■■■□□□□□□■□□□□□■■■□■■■ | 777737770040731 | 1 | 0,1 | 1 |  |  |  |  |  |  |
| 281 | NA | LAM10 *^0,94^ | ■■■■■■■■■■■■■□■■■■■■■□□□□■■■■■■■□□□□■■■■■■■ | 777757703760771 | 1 | 0,1 | 1 |  |  |  |  |  |  |
| 282 | NA | LAM9 *^1,00^ | ■■■■■■■■■■■■■■□■■■■■□□□□■■■■■■■□□□□□□■■□□■■ | 777767607740311 | 1 | 0,1 |  | 1 |  |  |  |  |  |
| 283 | NA | LAM9 *^1,00^ | ■■■■■■■■■■■■■■□■■■■■□□□□■■■■■■■■□□□□□□□■■□□ | 777767607760060 | 1 | 0,1 |  | 1 |  |  |  |  |  |
| 284 | NA | LAM9 *^1,00^ | ■■■■■■■■■■■■■■□■■■■■□□□□■■■■■■■■□□□□□■□■■□□ | 777767607760260 | 1 | 0,1 |  | 1 |  |  |  |  |  |
| 285 | NA | Haarlem1*^1,00^ | ■■■■■■■■■■■■■■□■■■■■■■■□□□□□□□□□□□□□■■■□■■■ | 777767760000731 | 1 | 0,1 | 1 |  |  |  |  |  |  |
| 286 | NA | T1*^1,00^ | ■■■■■■■■■■■■■■□■■■■■■■■■■■■■□□■■□□□□■■■■■■■ | 777767777460771 | 1 | 0,1 |  |  |  | 1 |  |  |  |
| 287 | NA | T1*^1,00^ | ■■■■■■■■■■■■■■■□■■■■□■■■■■■■■■■■□□□□■■■■■■■ | 777773677760771 | 1 | 0,1 |  | 1 |  |  |  |  |  |
| 288 | NA | LAM8 *^1,00^ | ■■■■■■■■■■■■■■■■■■■■□□□□□□□□□□□□□□□□□□□■■■■ | 777777600000071 | 1 | 0,1 |  |  | 1 |  |  |  |  |
| 289 | NA | LAM9 *^1,00^ | ■■■■■■■■■■■■■■■■■■■■□□□□■□□■■■■□□□□□■■■■■■■ | 777777604740771 | 1 | 0,1 | 1 |  |  |  |  |  |  |
| 290 | NA | LAM9 *^1,00^ | ■■■■■■■■■■■■■■■■■■■■□□□□■□□■■■■■□□□□■■■■■■■ | 777777604760771 | 1 | 0,1 | 1 |  |  |  |  |  |  |
| 291 | NA | LAM9* ^1,00^ | ■■■■■■■■■■■■■■■■■■■■□□□□■■□■■■□□□□□□■■□■■■■ | 777777606700671 | 1 | 0,1 |  | 1 |  |  |  |  |  |
| 292 | NA | LAM9 *^1,00^ | ■■■■■■■■■■■■■■■■■■■■□□□□■■■■□■■□□□□□■■■□■■■ | 777777607540731 | 1 | 0,1 |  |  |  | 1 |  |  |  |
| 293 | NA | Haarlem1*^1,00^ | ■■■■■■■■■■■■■■■■■■■■□■■□□□□□□□□□□□□□□□□■■■■ | 777777660000071 | 1 | 0,1 |  |  |  | 1 |  |  |  |
| 294 | NA | Familia33 *^1,00^ | ■■■■■■■■■■■■■■■■■■■■□■■□■■■■■■■■□■■■■■■■■■■ | 777777667767771 | 1 | 0,1 |  |  |  | 1 |  |  |  |
| 295 | NA | Haarlem3 *^0,77^ | ■■■■■■■■■■■■■■■■■■■■□■■■■■■■■■□■□□□□■■□■■■■ | 777777677720671 | 1 | 0,1 |  |  |  | 1 |  |  |  |
| 296 | NA | Familia33 *^1,00^ | ■■■■■■■■■■■■■■■■■■■■■■■□■■□■■■■■■■■□□□□■■■■ | 777777766776071 | 1 | 0,1 | 1 |  |  |  |  |  |  |
| 297 | NA | T2 *^0,98^ | ■■■■■■■■■■■■■■■■■■■■■■■□■■■■■■■■□□□□■■■□□□□ | 777777767760700 | 1 | 0,1 |  | 1 |  |  |  |  |  |
| 298 | NA | Haarlem1*^1,00^ | ■■■■■■■■■■■■■■■■■■■■■■■■■□□□□□□■□□□□■□□■■■■ | 777777774020471 | 1 | 0,1 |  | 1 |  |  |  |  |  |
| 299 | NA | T1*^1,00^ | ■■■■■■■■■■■■■■■■■■■■■■■■■□□□■■■■□□□□■■■■■■■ | 777777774360771 | 1 | 0,1 |  |  |  | 1 |  |  |  |
| 300 | NA | Haarlem3 *^0,72^ | ■■■■■■■■■■■■■■■■■■■■■■■■■■■■■□□□□□□□□□□□■■■ | 777777777600031 | 1 | 0,1 | 1 |  |  |  |  |  |  |
| **Total** |  |  |  |  | **1.298** | **100** |  |  |  |  |  |  |  |
